# A Review of Current Methodologies for Regional Evapotranspiration Estimation from Remotely Sensed Data

**DOI:** 10.3390/s90503801

**Published:** 2009-05-19

**Authors:** Zhao-Liang Li, Ronglin Tang, Zhengming Wan, Yuyun Bi, Chenghu Zhou, Bohui Tang, Guangjian Yan, Xiaoyu Zhang

**Affiliations:** 1 Institute of Geographic Sciences and Natural Resources Research, Beijing 100101, China; E-Mails: trl_wd@163.com (R.T.); zhouch@lreis.ac.cn (C.H.Z.); tangbh@igsnrr.ac.cn (B.H.T.); 2 LSIIT, Bld Sebastien Brant, BP10413, 67412 Illkirch, France; E-Mail: biyuyun@sina.com (Y.Y.B.); 3 Graduate University of Chinese Academy of Sciences, Beijing, 100049, China; 4 ICESS, University of California, Santa Barbara, CA 93106, USA; E-Mail: wan@icess.ucsb.edu; 5 Institute of Agricultural Resources and Regional Planning, Beijing 100081, China; 6 Beijing Normal University, Beijing 100875, China; E-Mail: gjyan@bnu.edu.cn; 7 Shanxi University, Taiyuan 030006, China; E-Mail: zhang_xyhz@sxu.edu.cn

**Keywords:** remote sensing, evapotranspiration, methodology, review, temporal scaling

## Abstract

An overview of the commonly applied evapotranspiration (ET) models using remotely sensed data is given to provide insight into the estimation of ET on a regional scale from satellite data. Generally, these models vary greatly in inputs, main assumptions and accuracy of results, etc. Besides the generally used remotely sensed multi-spectral data from visible to thermal infrared bands, most remotely sensed ET models, from simplified equations models to the more complex physically based two-source energy balance models, must rely to a certain degree on ground-based auxiliary measurements in order to derive the turbulent heat fluxes on a regional scale. We discuss the main inputs, assumptions, theories, advantages and drawbacks of each model. Moreover, approaches to the extrapolation of instantaneous ET to the daily values are also briefly presented. In the final part, both associated problems and future trends regarding these remotely sensed ET models were analyzed to objectively show the limitations and promising aspects of the estimation of regional ET based on remotely sensed data and ground-based measurements.

## 1. Introduction

Generally speaking, evapotranspiration (ET) is a term used to describe the loss of water from the Earth's surface to the atmosphere by the combined processes of evaporation from the open water bodies, bare soil and plant surfaces, etc. and transpiration from vegetation or any other moisture-contaning living surface. Water in an entity or at an interface and the energy needed to convert liquid water to the vapor form, along with some mechanism to transport water from the land surface to the atmosphere, are prerequisites to ensure the occurrence of ET. Other factors affecting ET rates mainly include solar radiation, wind speed, vapor pressure deficit and air temperature, etc.

At the beginning of 21^st^ century, there may be no other environmental problems of more concern for humans than global climate change. Global warming, natural hazards and species extinctions, etc., are several dangerous situations that might happen if climate change occurs too rapidly. The Intergovernmental Panel on Climate Change (IPCC) was established by the World Meteorological Organization (WMO) and the United Nations Environment Program (UNEP) in 1988 (http://www.ipcc.ch/about/index.htm) to evaluate the risk of climate change caused by human activity. ET, which governs the water cycle and energy transport among the biosphere, atmosphere and hydrosphere as a controlling factor, plays an important role in hydrology, meteorology, and agriculture, such as in prediction and estimation of regional-scale surface runoff and underground water, in simulation of large-scale atmospheric circulation and global climate change, in the scheduling of field-scale field irrigations and tillage, etc. [1-2]. On a global basis, the mean ET from the land surface accounts for approximately 60% of the average precipitation. It is therefore indispensable to have reliable information on the land surface ET when natural hazards such as floods and droughts are predicted and weather forecasting and climate change modeling are performed [3]. However, land surface ET, which is as important as precipitation and runoff in the water cycle modeling, is one of the least understood components of the hydrological cycle. In recent years, except for a few industrialized countries, most countries have undergone an increase of water use due to their population and economic growth and expended water supply systems, while irrigation water use accounts for about 70% of water withdrawals worldwide and for more than 90% of the water consumption and irrigation water use has been believed to be the most important cause of the increase of water use in most countries [4]. Estimation of water consumption based on ET models using remotely sensed data has become one of the hot topics in water resources planning and management over watersheds due to the competition for water between trans-boundary water users [5]. In climate dynamics, continuous progress has been made to describe the general circulation of the atmosphere and Brutsaert [3] has shown that the general circulation models appeared to be quite sensitive to the land surface ET information. For vegetated land surfaces, ET rates are closely related to the assimilation rates of plants and can be used as an indicator of plant water stress [6]. Therefore, accurate estimates of regional ET in the land surface water and energy budget modeling at different temporal and spatial scales are essential in hydrology, climatology and agriculture.

In various practical applications, there are still no specific ways to directly measure the actual ET over a watershed [3]. Conventional ET estimation techniques (i.e., pan-measurement, Bowen ratio, eddy correlation system, and weighing lysimeter, scintillometer, sap flow) are mainly based on site (field)-measurements and many of those techniques are dependent on a variety of model complexities. Though they can provide relatively accurate estimates of ET over a homogeneous area, conventional techniques are of rather limited use because they need a variety of surface accessory measurements and land parameters such as air temperature, wind speed, vapor pressure at a reference height, surface roughness, etc., which are difficult to obtain over large-scale terrain areas and have to be extrapolated/interpolated to various temporal and spatial scales with limited accuracy in order to initialize/force those models [1]. Remote sensing technology is recognized as the only viable means to map regional- and meso-scale patterns of ET on the Earth's surface in a globally consistent and economically feasible manner and surface temperature helps to establish the direct link between surface radiances and the components of surface energy balance [7-14]. Remote sensing technology has several marked advantages over conventional “point” measurements: 1) it can provide large and continuous spatial coverage within a few minutes; 2) it costs less when the same spatial information is required; 3) it is particularly practical for ungauged areas where man-made measurements are difficult to conduct or unavailable [15-16]. Remotely sensed surface temperature can provide a measure of surface from a resolution of a few cm^2^ from a hand-held thermometer to about several km^2^ from certain satellites [17]. Combining surface parameters derived from remote sensing data with surface meteorological variables and vegetation characteristics allows the evaluation of ET on local, regional and global-scales. Remote sensing information can provide spatial distribution and temporal evolution of NDVI (Normalized Difference Vegetation Index), LAI (Leaf Area Index), surface albedo from visible and near-infrared bands and surface emissivity and radiometric surface temperature from mid and thermal infrared bands, many of which are indispensable to most of the methods and models that partition the available energy into sensible and latent fluxes components [18]. The possibility and ability of using remote sensing technology to evaluate ET with the help of hand-held and airborne thermometers have been recognized and verified since 1970, but it was not until 1978 with the launch of HCMM (Heat Capacity Mapping Mission) and polar orbiting weather satellites-TIROS-N that data became available for such surface flux studies from spacecraft [19].

The potential of using thermal infrared data from space to infer regional and local scale ET has been extensively studied during the past 30 years and substantial progress has been made [20]. The methods vary in complexity from simplified empirical regressions to physically based surface energy balance models, the vegetation index-surface temperature triangle/trapezoid methods, and finally to data assimilation techniques, usually coupled with some numerical model that incorporates all sources of available information to simulate the flow of heat and water transfer through the soil-vegetation-atmosphere continuum [13]. In 1970s, when the split-window technique for surface temperature retrieval was not yet developed, ET evaluation was often accomplished by regressing thermal radiances from remote sensors and certain surface meteorological measurement variables (solar radiation, air temperature) to *in-situ* ET observations or by simulating a numerical model of a planetary boundary layer to continuously match the thermal radiances from satellites [1,19,21-22]. These methods and the refinements have been successfully used in mapping ET over local areas.

However, satellite remote sensing cannot provide near-surface variables such as wind speed, air temperature, humidity, etc., which has to a great extent limited the applications of the energy balance equation to homogeneous areas with uniform vegetation, soil moisture and topography [23]. Moreover, when compared to each other, approaches to deriving land surface ET differ greatly in model-structure complexity, in model inputs and outputs and in their advantages and drawbacks. Therefore, with the consideration of the characteristics of the various ET methods developed over the past decades and of the significance of land surface ET to hydrologists, water resources and irrigation engineers, and climatologists, etc., how to calculate the ET over a regional scale or how to estimate ET precisely based on the remote sensing technology has become a critical question in various ET-related applications and studies. Summaries and comparisons of different remote sensing-based ET approaches are urgently required and indispensable for us to better understand the mechanisms of interactions among the hydrosphere, atmosphere and biosphere of the earth.

This paper provides an overview of a variety of methods and models that have been developed to estimate land surface ET on a field, regional and large scales, based mainly on remotely sensed data. For each method or model, we shall detail the main theory and assumptions involved in the model development, and highlight its advantages, drawbacks and potential. In the latter part, methods of how to convert instantaneous ET to daily values, and the problems and issues are addressed. Nomenclature used in this paper is given in the Appendix.

## 2. Overviews of Remote Sensing-Based Evapotranspiration Models in the Past Decades

Generally, the commonly applied ET models using remote sensing data can be categorized into two types: (semi-)empirical methods and analytical methods. (Semi-)empirical methods are often accomplished by employing empirical relationships and making use of data mainly derived from remote sensing observations with minimum ground-based measurements, while the analytical methods involve the establishment of the physical processes at the scale of interest with varying complexity and requires a variety of direct and indirect measurements from the remote sensing technology and ground-based instruments.

### 2.1. Simplified Empirical Regression Method

The main theory of the simplified empirical regression method first proposed by Jackson *et al.* [22] in their study of irrigated wheat at Phoenix, Arizona (U.S.A.) directly relates the daily ET to the difference between instantaneous surface temperature (T_s_) and air temperature (T_a_) measured near midday at about 13h 30′ to 14h 00′ local time over diverse surfaces with variable vegetation cover [24]. The most general form of the simplified regression method can be expressed mathematically as:

(1) $$LE_{d}=R_{nd}-B{\left(T_{s}-T_{a}\right)}^{n}$$

where B and n are site-specific regression coefficients dependent on surface roughness, wind speed and atmospheric stability, etc. [20], which are determined either by linear least squares fit to data or by simulations based on a SVAT (Soil-Vegetation-Atmosphere Transfer) model [25] or on a boundary layer model [26].

The simplified regression method proposed by Jackson *et al.* and its refinements have attracted great attention in the subsequent operational applications of ET mapping. For example, they first demonstrated that the value of parameter B was 0.064 and n was unity by regressing daily ET data from a lysimeter to the daily net radiation and one-time measurement of (T_s_-T_a_), while Seguin *et al.* [27] regressed data over large homogeneous areas in France with regression coefficients of B = 0.025 and n = 1. Seguin and Itier [20] discussed the theoretical basis and applications of the simplified regression method proposed by Jackson *et al.* in [22], and showed that surface roughness, wind speed and atmospheric stability were the main contributing factors to the regression coefficients and finally recommended different sets of parameters of B and n applicable to ‘medium rough’ surfaces for stable and unstable cases, respectively. Thus, the imposition of a single value of B and n may be unacceptable and specific values should be adjusted according to the broad range of surface roughness, wind speed and atmospheric stability [12]. Carlson *et al.* [25] theoretically analyzed the implications of the regression coefficients in the simplified equation. They defined B as an average bulk conductance for the daily integrated sensible heat flux and n as a correction for non-neutral static stability. A SVAT model was utilized to simulate the relationships between the coefficients B, n and the fractional vegetation cover (*Fr*) under variable surface roughness and geostrophic wind speed circumstances ranging from 2 to 30 cm and 1 to 8.5 m/s, respectively [25]. The resultant formulae are expressed as:

(2) B=0.0175+0.05Fr(±0.002)

(3) n=1.004−0.335Fr(±0.053)

This relationship is generally valid at a time period between 12 h 00′ and 14h 00′ when temperature varies slowly with time [25].

The height of measurement of T_a_ in the simplified equation (1) is also not specially specified. Consequently, Jackson *et al.* [22] used the height of 1.5 m as the measurement level of T_a_ while Seguin and Itier [20] utilized 2 m instead. Carlson and Buffum [26] found that the simplified equation might be more applicable to regional-scale ET estimations if the air temperature and wind speed were measured or evaluated at a level of 50 m, because at this level the meteorological variables are insensitive to the surface characteristics. They also suggested that a surface temperature rise (e.g., between 08h 00′ and 10h 00′ local time) in the morning obtained from Meteosat or GOES (Geostationary Operational Environmental Satellites) could replace the difference between surface and air temperature, in which the regression coefficients were highly sensitive to wind speed and surface roughness.

Two implicit assumptions in the simplified equation are that daily soil heat flux can be assumed to be negligible and instantaneous midday value of sensible heat flux can adequately express the influence of partitioning daily available energy into turbulent fluxes [24, 28]. Several papers have tested and verified this simple procedure to estimate daily ET under diverse atmospheric conditions and variable vegetation covers [12,20,22,25,26,29,30]. All the contributions to this work have shown that the error of the calculated daily ET is about 1 mm/day, which is sufficiently small to give reliable information about the water availability at a regional level [31].

The main advantage of this procedure, whose inputs include only one-time measurements of T_s_ and T_a_ near midday and the daily net radiation, is its simplicity. Thus, application of edit the simplified empirical equation is very convenient so long as these ground-based near midday meteorological measurements and one-time remotely sensed radiometric surface temperatures are available. However, the obvious weakness is that the need to determine the site-specific parameters B and n have more or less limited the applications of the simplified equation method over regional scales with variable surface conditions.

### 2.2 Residual Method of Surface Energy Balance

The residual method of surface energy balance between land and atmosphere can be divided into two categories: 1) single-source models [2,32-36] and 2) dual-source models [37-42].

#### 2.2.1 Single-Source Model

Surface energy balance governs the water exchange and partition of the surface turbulent fluxes into sensible and latent heat in the soil-vegetation-atmosphere continuum. The residual method of surface energy balance is one of the most widely applied approaches to mapping ET at different temporal and spatial scales. When heat storage of photosynthetic vegetation and surface residuals and horizontal advective heat flow are not taken into account, the one-dimensional form of surface energy balance equation at instantaneous time scale can be expressed numerically as:

(4) $$LE=R_{n}-G-H$$

Each of the three components of the energy balance equation, including *R_n_, G* and *H*, can be estimated by combining remote sensing based parameters of surface radiometric temperature and shortwave albedo from visible, near infrared and thermal infrared wavebands with a set of ground-based meteorological variables of air temperature, wind speed and humidity and other auxiliary surface measurements. These calculations based on single-source and dual-source energy balance models will be addressed in the following sections.

##### 2.2.1.1. Net Radiation Equation (*Rn*)

Surface net radiation (*R_n_*) represents the total heat energy that is partitioned into *G, H* and *LE*. It can be estimated from the sum of the difference between the incoming (*R_s_*) and the reflected outgoing shortwave solar radiation (0.15 to 5 μm), and the difference between the downwelling atmospheric and the surface emitted and reflected longwave radiation (3 to 100 μm), which can be expressed as [10, 13]:

(5) $$R_{n}=(1-\alpha_{s})R_{s}+\varepsilon_{s}\varepsilon_{a}\sigma T_{a}^{4}-\varepsilon_{s}\sigma T_{s}^{4}$$

where *α_s_* is surface shortwave albedo, usually calculated as a combination of narrow band spectral reflectance values in the bands used in the remote sensing [43], *R_s_* is determined by a combined factors of solar constant, solar inclination angle, geographical location and time of year, atmospheric transmissivity, ground elevation, etc. [36], *ε_s_* surface emissivity evaluated either as a weighted average between bare soil and vegetation [44] or as a function of NDVI [33], *ε_a_* is atmospheric emissivity estimated as a function of vapor pressure [45].

Kustas and Norman [13] reviewed the uncertainties of various methods of estimating the net shortwave and longwave radiation fluxes and found that a variety of remote sensing methods of surface net radiation estimation had an uncertainty of 5-10% compared with ground-based observations on meteorologically temporal scales. Bisht *et al.* [46] proposed a simple scheme to calculate the instantaneous net radiation over large heterogeneous surfaces for clear sky days using only land and atmospheric products obtained using remote sensing data from MODIS-Terra satellite over Southern Great Plain (SGP). Allen *et al.* [36] detailed an internalized calibration model for calculating ET as a residual of the surface energy balance from remotely sensed data when surface slope and aspect information derived from a digital elevation model were taken into account.

##### 2.2.1.2. Soil Heat Flux (*G*)

Soil heat flux (*G*) is the heat energy used for warming or cooling substrate soil volume. It is traditionally measured with sensors buried beneath the surface soil and is directly proportional to the thermal conductivity and the temperature gradient with depth of the topsoil. The one used in SEBAL (Surface Energy Balance Algorithm for Land) [47] to estimate the regional-scale *G* is expressed as follows:

(6) $$G=0.30(1-0.98{\text{NDVI}}^{4})R^{n}$$

As *G* varies considerably from dry bare soil to highly well watered vegetated areas, it is inappropriate to extrapolate ground-based measurements to values of areal areas. Under current circumstances, it is still impossible to directly measure *G* from remote sensing satellite platforms. Fortunately, the magnitude of *G* is relatively small compared to *R_n_* at the daytime overpass time of satellites. Estimation error of *G* will thus have a small effect on the calculated latent heat flux. Many papers have found that the ratio of *G* to *R_n_* ranges from 0.05 for full vegetation cover or wet bare soil to 0.5 for dry bare soil [10,13,44,48-51] and this ratio is simply related in an exponential form to LAI [52], NDVI [11,36,47], T_s_ [36,53] and solar zenith angle [54] based on field observations. The value of *G* has been shown to be variable in both diurnal and yearly cycle over diverse surface conditions [55]. However, the assumption that daily value of *G* is equal to 0 and can be negligible in the daily energy balance is generally regarded as a good approximation [48]. Comparisons of *G* between results from these simplified techniques and observations at micrometeorological scales showed an uncertainty of 20-30% [13].

##### 2.2.1.3. Sensible Heat Flux (*H*)

The sensible heat flux (*H*) is the heat transfer between ground and atmosphere and is the driving force to warm/cool the air above the surface. In the single-source energy balance model, it can be calculated by combining the difference of aerodynamic and air temperatures with the aerodynamic resistance (*r_a_*) from:

(7) $$H=\rho c_{p}(T_{\text{aero}}-T_{a})/r_{a}$$

Aerodynamic resistance *r_a_* is affected by the combined factors of surface roughness (vegetation height, vegetation structure), wind speed and atmospheric stability, etc. Therefore aerodynamic resistance to heat transfer must be adjusted according to different surface characteristics except when the water is freely available [56]. Hatfield *et al.* [57] have shown that *r_a_* decreased as the wind speed increased, regardless of whether the surface was warmer or cooler than air, and *r_a_* decreased if the surface become rougher [57]. Various methods for calculating *r_a_* have been developed ranging from extremely elementary (a function of wind speed only) to quite rigorous ones (accounting for atmospheric stability, wind speed, surface “aerodynamic” roughness, etc.) [17, 27, 58-60], with the commonly applied one being [61]:

(8) $$r_{a}=\frac{\ln[(z_{a}-d)/z_{om}-\psi_{1}]\ln[(z_{a}-d)/z_{oh}-\psi_{2}]}{k^{2}u},$$

with neutral stability, *ψ* = *ψ* = 0.

Jackson *et al.* [62] found that *T_s_-T_a_* varied from -10 to +5 °C under medium to low atmospheric humidity, which shows that neutral stability cannot prevail under a wide range of vegetation cover and soil moisture conditions. Under stable and unstable atmospheric stability conditions, the Monin-Obukhov length [63] was introduced to measure the stability and it needs to be solved with *H* iteratively [51]:

(9) $$\Lambda =\frac{u{*}^{3}\rho c_{p}T_{a}}{\text{kgH}}$$

where if Λ < 0, unstable stability; Λ < 0, stable stability.

For unstable conditions (usually prevailing at daytime) with no predominant free convection, *ψ*_1_ and *ψ*_2_ can be expressed as [64]:

(10) $$\psi_{1}=2\ln(\frac{1+x}{2})+\ln(\frac{1+x^{2}}{2})-2\arctan(x)+\frac{\pi }{2}$$

(11) $$\psi_{2}=2\ln(\frac{1+x^{2}}{2})$$

(12) $$\text{with}\;x={\left(1-16\frac{z_{a}-d}{\Lambda }\right)}^{0.25}$$

For stable conditions (usually prevailing at night-time), the formula proposed by Webb [65] and Businger *et al.* [66] was adopted to account for the effects of atmospheric stability on *r_a_* :

(13) $$\psi_{1}=\psi_{2}=-5\frac{z_{a}-d}{\Lambda }$$

Hatfield *et al.* [57] have shown that ET rates could be over-estimated when the canopy-air temperature difference is greater than about ±2 °C, if the aerodynamic resistance is not corrected for atmospheric stability.

The surface roughness plays a significant role in the determination of sensible heat flux and it changes apparently with leaf size and the flexibility of petioles and plant stems [10]. The effective roughness for momentum *z_om_* is considered to be some unspecified distance above a zero plane displacement height where the wind speed is assumed to be zero when log-profile wind speed is extrapolated downward, rather than at true ground surface [67]. Some papers have specified *z_om_* is equal to *z_oh_* and can be either a function of vegetation height [68-69], in which *z_om_* is typically 5 to 15 percent of vegetation height depending on vegetation characteristics [70], or estimated from wind profiles, using an extrapolation of the standard log-linear wind relationship to zero wind speed [69]. Brutsaert [61] showed that the heat transfer was mainly driven by molecular diffusion while the momentum transfer near the surface was controlled by both viscous shear and pressure forces. Because of the differences between heat and momentum transfer mechanisms, there is a distinction between *z_om_* and *z_oh_*, which has caused an additional resistance (often expressed as aerodynamic definition of *kB^-1^* : kB^-1^ = ln(*z_om_/z_oh_*) [44]) to heat transfer [71] or an excess (extra) resistance [72]. Stewart *et al.* [73] have related the excess resistance to the dimensionless bulk parameter *kB^-1^* using the following expression:

(14) $$r_{e}=\frac{kB^{-1}}{ku^{*}}$$

Verhoef *et al.* [74] showed that *kB^-1^* was sensitive to measurement errors both in the micrometeorological variables and in the roughness length for momentum and its value over bare soil could be less than zero. Massman [75] used a physically based “localized near-field” Lagrangian theory to evaluate the effects of *kB^-1^* on the vegetative components in the two-source energy balance models and on the combined effects of soil and vegetation in a single-source model. Su *et al.* [76] proposed a quadratic weighting based on the fractional coverage of soil and vegetation to calculate the *kB^-1^* in order to take into account any situation from full vegetation to bare soil conditions. What should be noted is that the determination of the surface roughness still remains a challenging issue for large scale retrieval of the turbulent heat fluxes in spite of the efforts made in the past.

Klaassen and van den Berg [77] showed that the measurement (or reference) height should be set at 50 m instead of 2 m at the bottom of the mixed layer and calculation of ET of crops over rough surfaces could be improved with increasing reference height.

*T_aero_*, the temperature at level of *d*+*z_oh_*, which is the average temperature of all the canopy elements weighted by the relative contribution of each element to the overall aerodynamic conductance [11], may be estimated from extrapolation of temperature profile down to *z* = *d*+*z_oh_* and is recognized as the temperature of the apparent sources or sinks of sensible heat [78]. A number of papers have utilized remotely sensed surface temperature *T_s_* instead of *T_aero_* in Equation (7) to calculate *H* over a wide range of vegetated surfaces because *T_aero_* is very difficult to measure [11,27,78-79]. However, there are problems associated with the assumption that measured *T_s_* is identical to *T_aero_* [78]. *T_aero_* is found to be higher (lower) than *T_s_* under stable (unstable) atmospheric conditions and they are nearly the same under neutral conditions [78]. Kustas and Norman [13] concluded that the differences between *T_aero_* and *T_s_* could range from 2 °C over uniform vegetation cover to 10 °C for partially vegetated areas. Subsequently dual-source (two-source) models have been developed to account for the differences between *T_aero_* and *T_s_*, and thus avoid the needs for adding excess resistance in Equation (7) [37].

The bulk transfer equation (resistance-based model) expressed in Equation (7) has been predominately applied since 1970s over a local/regional scale with various vegetation covers [17, 79-80]. The average difference of *H* estimated by different authors based on the bulk transfer equation is about 15-20%, which is around the magnitude of uncertainty in eddy correlation and Bowen ratio techniques for determining the surface fluxes in heterogeneous terrain [13,56].

Generally speaking, energy balance models are theoretically verified and physically based. Single source models are usually computationally timesaving and require less ground-based measurements, compared to dual-source models. Over homogeneous areas, single-source models can evaluate ET with a relatively high accuracy. But over partially vegetated areas, there is a strong need to develop a dual-source model to separately model the heat and water exchange and interaction between soil and atmosphere and between vegetation and atmosphere, which often deals with a decomposition of radiometric surface temperature to soil and vegetation component temperatures either from multi-angular remotely sensed thermal data or from an iteration of respective solution of soil and vegetation energy balance combined with a Priestly-Taylor equation. A major dilemma with both the physics-based single and dual-source models lies in the requirements for sufficiently detailed parameterization of surface soil and vegetation properties and ground-based measurements, such as air temperature, wind speed, surface roughness, vegetation height, etc., as model inputs.

In the single-source surface energy balance models, the main distinction of various methods is how to estimate the sensible heat flux. Some of them are based on the spatial context information (emergence of representative dry and wet pixels) of land surface characteristics in the area of interest. Some of them are not. Below we will review several representative single-source energy balance models.

###### (1) SEBI (Surface Energy Balance Index) and SEBS (Surface Energy Balance System)

SEBI, first proposed by Menenti and Choudhury [81], along with its derivatives like SEBAL, S-SEBI (Simplified-SEBI), SEBS, METRIC (Mapping EvapoTranspiration at high Resolution with Internalized Calibration) etc., are typically single-source energy balance models based on the contrast between dry and wet limits to derive pixel by pixel ET and EF from the relative evaporative fraction when combined with surface parameters derived from remote sensing data and a certain amount of ground-based variables measured at local and/or regional scale [82]. The dry (wet) limit, no matter how it was specifically defined, often has the following characteristics: 1) generally maximum (minimum) surface temperature, 2) usually low or no (high or maximum) ET.

In the SEBI method, the dry limit is assumed to have a zero surface ET (latent heat flux) for a given set of boundary layer characteristics (potential temperature, wind speed and humidity, etc.). So the sensible heat flux is then equal to the surface available energy, with the *T*_*s*,max_ inverted from the bulk transfer equation being expressed as [83]:

(15) $$T_{s,\max}=T_{\text{pbl}}+r_{a,\max}\frac{H}{\rho c_{p}}$$

Correspondingly, the minimum surface temperature can be evaluated from the wet limit, where the surface is regarded as to evaporate potentially and the potential ET is calculated from Penman-Monteith equation with a zero internal-resistance. The *T*_*s*,min_ is expressed as [83]:

(16) $$T_{s,\min}=T_{\text{pbl}}+\frac{r_{a,\min}\frac{R_{n}-G}{\rho c_{p}}-\text{VPD}/\gamma }{1+\Delta /\gamma }$$

The relative evaporation fraction can then be calculated by interpolating the observed surface temperature within the maximum and minimum surface temperature in the following form [83]:

(17) $$\frac{LE}{LE_{p}}=1-\frac{{r_{a}}^{-1}(T_{s}-T_{\text{pbl}})-{r_{a,\min}}^{-1}(T_{s,\min}-T_{\text{p}bl})}{{r_{a,\max}}^{-1}(T_{s,\max}-T_{\text{pbl}})-{r_{a,\min}}^{-1}(T_{s,\min}-T_{\text{pbl}})}$$

where the second part of the right hand side of Equation (17) is the so-called SEBI, which varies between 0 (actual = potential ET) and 1 (no ET).

Parameterization of the SEBI approach was first proposed by defining theoretical pixel-wise ranges for *LE* and *T_s_* to account for spatial variability of actual evaporation due to albedo and aerodynamic roughness [81]. This parameterization was essentially a modification from CWSI (Crop Water Stress Index) proposed by Idso *et al.* [84] and Jackson *et al.* [6,85]. The theoretical CWSI accounted for the effects of the net radiation and wind speed in addition to the temperature and vapor pressure required by the empirical CWSI.

Taking into account the dependence of external resistance on the atmospheric stratification, Menenti and Choudhury [81] proposed an approach to calculate the pixel-wise maximum and minimum surface temperature and redefined CWSI as a pixel-wise SEBI at given surface reflectance and roughness to derive the regional ET from the relative evaporative fraction. The CWSI was based on surface meteorological scaling while the SEBI used planetary boundary layer scaling.

Subsequently the SEBAL, SEBS and S-SEBI models have been developed based on this conception of SEBI. The main distinction between each of these models and other commonly applied single-source models is the difference in how they calculate the sensible heat flux or precisely how to define the dry (maximum sensible heat and minimum latent heat) and wet (maximum latent heat and minimum sensible heat) limits and how to interpolate between the defined upper and lower limits to calculate the sensible heat flux for a given set of boundary layer parameters of both remotely sensed *T_s_*, albedo, NDVI, LAI, *F_r_* and ground-based air temperature, wind speed, humidity, vegetation height, etc. Assumptions in SEBI, SEBAL, S-SEBI, SEBS models are that there are few or no changes in atmospheric conditions (mainly the surface available energy) in space and sufficient surface horizontal variations are required to ensure dry and wet limits existed in the study region.

The Surface Energy Balance System (SEBS), detailed by Su [2,76] and Su *et al.* [86,87] with a dynamic model for the thermal roughness and the Bulk Atmospheric Similarity theory for PBL scaling and the Monin-Obukhov Atmospheric Surface Layer (ASL) similarity for surface layer scaling, is an extension from the concept of SEBI for the estimation of land surface energy balance using remotely sensed data in a more complex framework. SEBS consists of: 1) a set of tools for the calculations of land surface physical parameters; 2) calculation of roughness length for heat transfer; 3) estimation of the evaporative fraction based on energy balance at limiting cases [2]. In SEBS, at the dry limit, latent heat flux is assumed to be zero due to the limitation of soil moisture which means sensible heat flux reaches its maximum value (i.e., *H_dry_* = *R_n_-G*). At the wet limit, ET takes place at potential rate (*LE_wet_*), (i.e. ET is limited only by the energy available under the given surface and atmospheric conditions, which can be calculated by a combination equation similar to the Penman-Monteith combination equation [88] assuming that the bulk internal resistance is zero), the sensible heat flux reaches its minimum value, *H_wet_*. The sensible heat flux at dry and wet limits can be expressed as:

(18) $$H_{\text{dry}}=R_{n}-G$$

(19) $$H_{\text{wet}}=((R_{n}-G)-\frac{\rho C_{p}}{r_{a}}\frac{\text{VPD}}{\gamma })/(1+\frac{\Delta }{\gamma })$$

where *r_a_* is dependent on the Obukhov length, which in turn is a function of the friction velocity and sensible heat flux.

The *EF_r_* and *EF* then can be expressed as:

(20) $$EF_{r}=1-\frac{H-H_{\text{wet}}}{H_{\text{dry}}-H_{\text{wet}}}$$

(21) $$EF=\frac{EF_{r}\cdot LE_{\text{wet}}}{R_{n}-G}$$

*H* can be solved using a combination of a dynamic model for thermal roughness [76] and the Bulk Atmospheric Similarity theory of Brutsaert [89] for Planetary Boundary Layer scaling and the Monin-Obukhov Atmospheric Surface Layer similarity for surface layer scaling [63].

In SEBS, distinction is made between the ABL (Atmospheric Boundary Layer) or PBL (Planetary Boundary Layer) and the ASL similarity. Inputs to the SEBS include remote sensing data-derived land parameters and ground-based meteorological measurements, such as land surface temperature, LAI, fractional vegetation cover, albedo, wind speed, humidity, air temperature. Jia *et al.* [90] described a modified version of SEBS using remote sensing data from ATSR and ground data from a Numerical Weather Prediction model and validated the estimated sensible heat flux with large aperture scintillometers located at three sites in Spain. With the surface meteorology derived from the Eta Data Assimilation System, Wood *et al.* [91] applied SEBS to the SGP region of the United States where the ARM (Atmospheric Radiation Measurement) program had been carried out by the U.S. Department of Energy. Derived latent heat fluxes were compared with the measurements from the EBBR sites and results indicated that the SEBS approach had promise in estimating surface heat flux from space for data assimilation purposes. SEBS has been used to estimate daily, monthly and annual evaporation in a semi-arid environment [86]. Su [2] showed that SEBS could be used for both local scaling and regional scaling under all atmospheric stability regimes. Results from Su *et al.* [92] have shown that accuracy of ET value estimated from SEBS could reach 10-15% of that of *in-situ* measurements collected during the Soil Moisture-Atmosphere Coupling Experiment even when evaporative fraction ranged from 0.5 to 0.9.

Advantages of the SEBS are that: 1) uncertainty from the surface temperature or meteorological variables in SEBS can be limited with consideration of the energy balance at the limiting cases; 2) new formulation of the roughness height for heat transfer is developed in SEBS instead of using fixed values; 3) *a priori* knowledge of the actual turbulent heat fluxes is not required. However, too many required parameters and relatively complex solution of the turbulent heat fluxes in SEBS can be the source of more or less inconveniences when data are not readily available.

###### (2) S-SEBI

A new method, called S-SEBI and developed by Roerink *et al.* [34] to derive the surface energy balance, has been tested and validated with data from a small field campaign conducted during August 1997. The main theory of S-SEBI is based on the contrast between a reflectance (albedo) dependent maximum surface temperature for dry limit and a reflectance (albedo) dependent minimum surface temperature for wet limit to partition available energy into sensible and latent heat fluxes.

**Figure 1.**
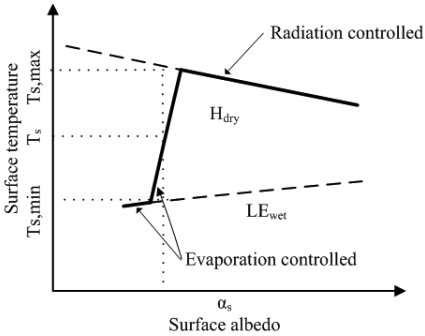
Theoretically schematic relationship between surface temperature and albedo in the S-SEBI (after [34]).

A theoretical explanation to S-SEBI, when a wide range of surface characteristics changing from dry/dark soil to wet/bright pixels exist, can be given: 1) at low reflectance (albedo), surface temperature keeps almost unchangeable because of the sufficient water available under these conditions, such as over open water or irrigated lands; 2) at higher reflectance (albedo), surface temperature increases to a certain point with the increases of reflectance due to the decrease of ET resulting from the less water availability, which is termed as “evaporation controlled”; 3) after the inflexion, the surface temperature will decrease with the increases of surface reflectance (albedo), which is called the “radiation controlled” (see Figure 1).

In S-SEBI, the evaporative fraction is bounded by the dry and wet limits and formulated by interpolating the reflectance (albedo) dependent surface temperature between the reflectance (albedo) dependent maximum surface temperature and the reflectance (albedo) dependent minimum surface temperature, which can be expressed as:

(22) $$EF=\frac{T_{s,\max}-T_{s}}{T_{s,\max}-T_{s,\min}}$$

Where *T_s,max_* corresponds to the minimum latent heat flux (*LE_dry_* = 0) and maximum sensible heat flux (*H_dry_* = *R_n_-G*) [the upper decreasing envelope when *T_s_* is plotted against surface reflectance (albedo)], *T_s,min_* is indicative of the maximum latent heat flux (*LE_wet_* = *R_n_-G*) and minimum sensible heat flux (*H_wet_* = 0) (the lower increasing envelope when *T_s_* is plotted against surface reflectance). *T_s,max_* and *T_s,min_* are regressed to the surface reflectance (albedo):

(23) $$T_{s,\max}=a_{\max}+b_{\max}\alpha_{s}$$

(24) $$T_{s,\min}=a_{\min}+b_{\min}\alpha_{s}$$

where *a_max_, b_max_, a_min_* and *b_min_* are empirical coefficients estimated from the scatter plot of *Ts* and *α_s_* over study area.

Inserting Equations (23-24) into Equation (22), *EF* can be derived by:

(25) $$EF=\frac{a_{\max}+b_{\max}\alpha_{s}-T_{s}}{a_{\max}-a_{\min}+(b_{\max}-b_{\min})\alpha_{s}}$$

If the atmospheric conditions over the study area can be regarded as constant and sufficient variations in surface hydrological conditions are present, the turbulent fluxes then can be calculated with S-SEBI without any further information than the remote sensing image itself. Results from Roerink *et al.* [34] have shown that measured and estimated evaporative fraction values had a maximum relative difference of 8% when measurements obtained from a small field campaign during 1997 in Italy were compared with the S-SEBI derived outputs. Accuracy for the daily evapotranspiration using the S-SEBI method was found to be lower than 1 mm/d over a barrax test site in the framework of the DAISEX (Digital Airborne Imaging Spectrometer Experiment) campaigns [93]. Sobrino *et al.* [94] used the S-SEBI model with AVHRR data acquired from 1997 to 2002 over the Iberian Peninsula to analyze the seasonal evolution of daily ET and a RMSE of 1.4 mm/d has been shown when results derived from S-SEBI were checked against with high resolution ET values. Good results inferred from S-SEBI have been also reported by several other authors in different parts of the world [95-96].

The major advantages of this S-SEBI are that: 1) besides the parameters of the surface temperature and reflectance (albedo) derived from remote sensing data no additional ground-based measurement is needed to derive the EF if the surface extremes are present in the remotely sensed imagery; 2) the extreme temperatures in the S-SEBI for the wet and dry conditions vary with changing reflectance (albedo) values, whereas other methods like SEBAL try to determine a fixed temperature for wet and dry conditions. However, it should be noted that atmospheric corrections to retrieve *Ts* and *α_s_* from satellite data and determination of the extreme temperatures for the wet and dry conditions are location-specific when atmospheric conditions over larger areas are not constant any more.

###### (3) SEBAL and METRIC

SEBAL, developed by Bastiaanssen [97] and Bastiaanssen *et al.* [33] to evaluate ET with minimum ground-based measurements, has been tested at both field and catchment scales under several climatic conditions in more than 30 countries worldwide, with the typical accuracy at field scale being 85% and 95% at daily and seasonal scales, respectively [5,47,53,98].

One of the main considerations in SEBAL, when evaluating pixel by pixel sensible and latent heat fluxes, is to establish the linear relationships between *T_s_* and the surface-air temperature difference *dT* on each pixel with the coefficients of the linear expressions determined from the extremely dry (hot) and wet (cold) points. The *dT* can be approximated as a relatively simple linear relation of *T_s_* expressed as:

(26) $$dT=a+bT_{s}$$

where *a* and *b* are empirical coefficients derived from two anchor points (dry and wet points).

At the dry (hot) pixel, latent heat flux is assumed to be zero and the surface-air temperature difference at this pixel is obtained by inverting the single-source bulk aerodynamic transfer equation:

(27) $$dT_{\text{dry}}=\frac{H_{\text{dry}}\times r_{a}}{\rho C_{p}}$$

where *H_dry_* is equal to *R_n_-G*.

At the wet (cold) pixel, latent heat flux is assigned a value of *R_n_-G* (or a reference ET), which means sensible heat flux under this condition is equal to zero (when reference ET is applied, both *H* and *dT* at this pixel will not equal zero any more). Obviously, the surface-air temperature difference at this point is also zero (*dT_wet_* = 0).

After calculating surface-air temperature differences at both dry (hot) and wet (cold) points, coefficients *a* and *b* in Equation (26) can be obtained. Providing that *a* and *b* are known, the surface-air temperature difference *dT* at each pixel over the study area is estimated with *T_s_* using Equation (26). Finally, *H* can be obtained iteratively with *r_a_* corrected for stability using Equation (7). This procedure requires wind speed measured at ground to be extrapolated to a blending height of about 100 to 200 m where wind speed at this level is assumed to not be affected by surface variations.

SEBAL has been applied for ET estimation, calculation of crop coefficients and evaluation of basin wide irrigation performance under various agro-climatic conditions in several countries including Spain, Sri Lanka, China, and the United States [5, 99]. Timmermans *et al.* [100] compared the spatially distributed surface energy fluxes derived from SEBAL with a dual-source energy balance model using data from two large scale field experiments covering sub-humid grassland (Southern Great Plains '97) and semi-arid rangeland (Monsoon '90). Norman *et al.* [101] showed that the assumption of linearity between surface temperature and the air temperature gradient used in defining the sensible heat fluxes did not generally hold true for strongly heterogeneous landscape. Teixeira *et al.* [102-103] reviwed the inputs to SEBAL model and assessed ET and water productivity with SEBAL using ground measurements observed over the semi-arid region of the Low-Middle São Francisco River basin, Brazil. Opoku-Duah *et al.* [104] employed the SEBAL model with remote sensing data derived respectively from MODIS and AATSR sensors to estimate ET over large heterogeneous landscapes and found that both sensors underestimated daily ET when compared with eddy correlation observations. The selection of dry pixel and wet pixel can have a significant impact on the heat flux distribution from SEBAL.

One of the assumptions made in the SEBAL model is that full hydrological contrast (i.e., wet and dry pixels) is presented in the area of interest. The most key aspect in the SEBAL is to identify the dry pixels while wet pixels are often determined over a relatively large calm water surface or at a location of well-watered areas. The advantages of the SEBAL over previous approaches to estimate land surface fluxes from thermal remote sensing data are: 1) it requires minimum auxiliary ground-based data; 2) it does not require a strict correction of atmospheric effects on surface temperature thanks to its automatic internal calibration, and 3) internal calibration can be done within each analyzed image. However, SEBAL has several drawbacks: 1) it requires subjective specifications of representative hot/dry and wet/cool pixels within the scene to determine model parameters *a* and *b*; 2) it is often applied over flat surfaces. When SEBAL is applied over mountainous areas, adjustments based on a digital elevation model need to be made to *Ts* and *u* to account for the lapse rate; 3) errors in surface temperatures or surface-air temperature differences have great impacts on H estimate; 4) radiometer viewing angle effects, which can cause variation in *T_s_* of several degrees for some scenes, have not been taken into account.

To avoid the limitations of the SEBAL in mapping regional ET over more complicated surfaces, Allen *et al.* [105-107,36] highlighted a similar SEBAL-based approach, named METRIC, to derive ET from remotely sensed data in the visible, near-infrared and thermal infrared spectral regions along with ground-based wind speed and near surface dew point temperature. In METRIC, an automatic internal calibration method similar to SEBAL (linearly relating *T_s_* to the surface-air temperature difference) is used to calculate the sensible and latent heat fluxes.

Gowda *et al.* [108] have evaluated the performance of the METRIC model in the Texas High Plains using Landsat 5 TM data acquired on two different days in 2005 by comparison of resultant daily ET with measured values derived from soil moisture budget. Santos *et al.* [109] have found that combing a water balance model with ET estimated from METRIC model could provide significant improvements in the irrigation schedules in Spain. Tasumi *et al.* [110] found that SEBAL/METRIC models had high potential for successful ET estimates in the semi-arid US by comparing the derived ET with lysimeter-measured values.

Main distinctions between METRIC and SEBAL are: 1) METRIC does not assume *H_wet_* = 0 or *LE_wet_* = *R_n_-G* at the wet pixel, instead a daily surface soil water balance is run to confirm that for the hot pixel, ET is equal to zero, and for the wet pixel, ET is set to 1.05 ET_r_, where ET_r_ is the hourly (or shorter time interval) tall reference (like alfalfa) ET calculated using the standardized ASCE Penman-Monteith equation; 2) wet pixels in METRIC are selected in an agricultural setting where the cold pixels should have biophysical characteristics similar to the reference crop (alfalfa); 3) the interpolation (extrapolation) of instantaneous ET to daily value is based on the alfalfa ET_r_F (defined as the ratio of instantaneous ET to the reference ET_r_ that is computed from meteorological station data at satellite overpass time) instead of the actual evaporative fraction, which can better account for the impacts of advection and changing wind and humidity conditions during the day.

###### (4) VI-Ts Triangle/Trapezoidal Feature Space

The *VI-T_s_* triangle feature space, derived from the contextual information of remotely sensed surface temperature *Ts* and Vegetation Index (*VI*), was first proposed by Goward *et al.* [111], and subsequently was utilized to study the soil water content, surface resistance, land use and land cover change, drought monitoring and regional ET [112-120] while the trapezoidal space was derived from a simple CWSI [6,84].

**Figure 2.**
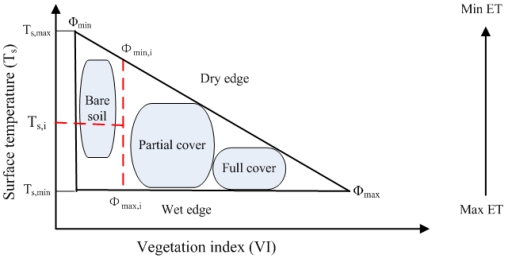
The simplified VI-T_s_ triangular space (after [133]).

The *T_s_-VI* triangle/trapezoidal feature space established under the conditions of full ranges of soil moisture content and vegetation is characteristic of being bounded with an upper decreasing envelope (dry edge, defined as the locus of the highest surface temperatures under differing amounts of vegetation cover at a given atmospheric forcing, which is assumed to represent pixels of unavailability of soil moisture content) and a lower nearly horizontal envelope (wet edge, defined as the locus of the lowest surface temperatures under differing amounts of vegetation cover, which is regarded to describe pixels in the potential ET at the given atmospheric forcing) with increasing vegetation cover and the two envelopes ultimately intersect at a (truncated) point at full vegetation cover (see Figure 2).

The principal rationale of the *T_s_-VI* triangle and trapezoid to be applied to evaluate ET at regional scale will be addressed respectively as follows.

####### i) Triangle Method

The simplicity of a Priestley-Taylor formulation with fully remotely sensed data proposed by Jiang and Islam [115-117] representatively based on the interpretations of the remotely sensed *T_s_*-NDVI triangle feature space, has been employed to estimate regional *EF* and ET, which can be expressed as:

(28) $$LE=\phi [(R_{n}-G)\frac{\Delta }{\Delta +\gamma }]$$

where *ϕ* ranges from 0 to 1.26. In Equation (28), all terms in the right-hand side can be calculated using remotely sensed data [115].

Solution of parameter *ϕ* in Equation (28) generally involves a certain degree of simplicity and some assumptions, including: 1) a complete range of soil moisture and vegetation coverage at satellite pixel scale should be ensured; 2) contaminations of clouds and atmospheric effects have to be removed; 3) two-step linear interpolation scheme [115,121-122] is used to get the value of *ϕ* in Equation (28) based on the *T_s_-NDVI* triangle feature space as displayed in Figure 2. This two-step linear interpolation is realized in the following manner: 1) a global minimum and maximum *ϕ* are respectively set to *ϕ*_min_ = 0 on the driest bare soil pixel and *ϕ*_max_ = 1.26 on the pixel with largest *NDVI* and lowest *T_s_*, and *ϕ*_min,*i*_ for each *NDVI* interval (*i*) is linearly interpolated with *NDVI* between *ϕ*_min_ and *ϕ*_max_, and *ϕ*_max,*i*_ for each *NDVI* (*i*) is calculated using the lowest surface temperature within that *NDVI* interval (generally, one assumes that *ϕ*_max,*i*_ = *ϕ*_max_ =1.26); 2) *ϕ_i_* within each *NDVI* interval is linearly increased with the decrease of Ts between *ϕ*_min,*i*_ and *ϕ*_max,*i*_.

The triangular (trapezoidal) feature space (*T_s_-VI*) constructed by plotting the remotely sensed surface temperature (or temperature difference, or a scaled surface temperature) against the vegetation indices (e.g., *NDVI, SAVI* - Soil-Adjusted Vegetation Index, a scaled *NDVI*, or *F_r_* - fractional vegetation cover) for a full range of variability in surface soil moisture and fractional vegetation cover has been found in a series of papers to derive surface soil moisture, and surface fluxes [25,51,60,111-113,123-129] and has been verified using measurements collected during the Monsoon '90 [130] and the FIFE 1987 and 1989 field programs [131]. Jiang and Islam [115] proposed the *NDVI-T_s_* triangle scheme to estimate surface ET over large heterogeneous areas from AVHRR data over the Southern Great Plain. The proposed approach appeared to be more reliable and easily applicable for operational estimate of ET over large areas. Gillies and Carlson [129] and Carlson [122] have examined the triangular patterns of *T_s_* plotted against *VI* using the simulated surface temperature and *NDVI* with a SVAT model on a theoretical basis and analyzed the spatial distributions of surface soil moisture availability and EF in the triangle feature space. Batra *et al.* [127] have analyzed the effects of spatial resolution of different remote sensing data on the *VI-T_s_* triangle with MODIS, NOAA16 and NOAA14 data in the Southern Great Plain in USA. Wang *et al.* [128] combined the advantages of both the thermal inertia method and the *T_s_-NDVI* spatial variation method to develop a day-night *T_s_* difference-NDVI approach and satisfactory results have been obtained at the Southern Great Plains of the United States from April 2001 to May 2005 when compared with the ground-based observations collected by Energy Balance Bowen Ratio Systems. The triangle method, proposed by Jiang and Islam [115], was modified by Stisen *et al.* [121] to take into account of the non-linear interpolation between *ϕ* and the surface temperature to estimate surface fluxes based entirely on remotely sensed data from MSG-SEVIRI sensor. Carlson *et al.* [25] have shown that the emergence of the triangle shape when the scatter plots of *T_s_* versus *VI* were plotted under the same coordinate system seemed to depend more on the number of pixels rather than just the spatial resolutions. Thus the triangle/trapezoid can be found from *T_s_* and *VI* data derived from satellites/sensors of different scales, such as the higher-resolution TM and the lower-resolution GOES data [132].

Implications in the so-called triangle/trapezoidal method are that: 1) the sensitivity of surface temperature to canopy and soil differs and canopy temperature is insensitive to surface/deep-layer soil moisture content, which contributes to the (truncated) vertex at full vegetation cover; 2) variations in the *VI-T_s_* triangle space are not primarily caused by differences in atmospheric conditions but by the variations in available soil water content.

The major assets of the remotely sensed *VI-T_s_* triangle method are that: 1) it allows for accurate estimate of regional ET with no auxiliary atmospheric or ground data besides the remotely sensed surface temperature and vegetation indices; 2) it is relatively insensitive to the correction of atmospheric effects. The limitations are that: 1) determination of the dry and wet edges requires a certain degree of subjectivity; 2) a large number of pixels over a flat area with a wide range of soil wetness and fractional vegetation cover are required to make sure that the dry and wet limits exist in the *VI-T_s_* triangle space.

####### ii) Trapezoid Method

**Figure 3.**
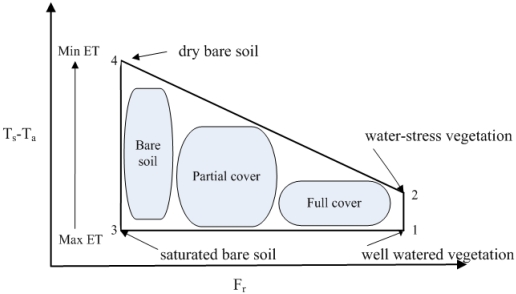
The hypothetical trapezoidal space between *T_s_-T_a_* and *F_r_* (after [60]).

On the basis of CWSI [6], Moran *et al.* [60] introduced a Water Deficit Index (*WDI*, defined as 1 minus the ratio of actual to potential ET) for ET estimatation based on the Vegetation Index/Temperature (*VIT*) trapezoid to extend the application of CWSI over fully to partially vegetated surface areas. The ground-based inputs to the trapezoid method include vapor pressure, air temperature, wind speed, maximum and minimum stomatal resistances, etc. One of the assumptions in the trapezoid approach is that values of *T_s_-T_a_* vary linearly with vegetation cover along crop extreme conditions edges while all the intermediary conditions relating *T_s_-T_a_* to a vegetation index are included within the constructed trapezoid. In order to calculate the *WDI* value of pixels of intermediate vegetation cover and soil moisture content for a specific time, four vertices of the trapezoid, corresponding to: (1) well watered full-cover vegetation; (2) water-stress full-cover vegetation; (3) saturated bare soil, and (4) dry bare soil, should be computed firstly combined with the CWSI theory and Penman-Monteith equation (see Figure 3). Moran *et al.* [60] defined/assumed the dry edge and wet edge respectively as the linear line connecting vertex (2) with vertex (4) and the linear line linking vertices between vertex (1) and vertex (3), as displayed in Figure 3. *WDI* within each *VI* from bare soil to full vegetation cover in the trapezoid is linearly related to the maximum and minimum temperature differences (*T_s_-T_a_*) and values of *WDI* equal to 0 and 1, respectively, correspond to minimum and maximum temperature differences. Therefore, for a partially vegetated surface, *WDI* can be defined as:

(29) $$\text{WDI}=1-LE/LE_{P}=[{\left(T_{s}-T_{a}\right)}_{\min}-{\left(T_{s}-T_{a}\right)}_{i}]/[{\left(T_{s}-T_{a}\right)}_{\min}-{\left(T_{s}-T_{a}\right)}_{\max}]$$

The trapezoid method is in essence an extension of CWSI developed by Idso *et al.* [84] and Jackson *et al.* [6]. CWSI is a commonly used index for detection of plant water stress based on the difference between canopy and air temperature and is only appropriate to apply for full-cover vegetated areas and bare soils at local and regional scales [60]. Idso *et al.* [84] proposed an empirical CWSI to quantify canopy stress by determining ‘non-water-stressed baselines’ for crops, in which the baselines represented the lower limit of the difference of canopy to air temperature when the plants are transpiring at the potential rate. Shortly, Jackson *et al.* [6, 85] defined the theoretical CWSI by ratioing the difference between the measured canopy temperature and the lower limit (corresponding to canopy transpiring potentially) to the difference between the upper (corresponding to non-transpiring canopy) and lower limits. The trapezoid method [(*T_s_-T_a_*)-*SAVI*] is a method to measure the surface water stress based on the formed trapezoid given a full range of surface vegetation cover and soil moisture content when the difference between surface and air temperature is plotted against a vegetation index [60, 125]. Kustas and Norman [13] have found that this trapezoid method permitted the concept of CWSI applicable to both heterogeneous and uniform areas and did not require the range of *VI* and surface temperature in the scene of interest as that proposed by Carlson *et al.* [134] and Price [124]. Luquet *et al.* [135] evaluated the impact of complex thermal infrared directional effects on the application of *WDI* using multidirectional crop surface temperatures and reflectance data acquired on a row-cotton crop with different water and cover conditions in Montpellier (France). Boulet *et al.* [136] found that the difference between actual and unstressed surface temperature is alomost linearly related to the water stress and is more relevant to detect second-stage processes than surface-air temperature difference even with inaccurate but realistic surface parameters. Results from the work of Moran *et al.* [60] showed that the *WDI* provided accurate estimates of field ET rates and relative field water deficit for both full cover and partially vegetated sites.

One of the advantages in the *VI-T_s_* trapezoidal space over the triangular space is that the *VI-T_s_* trapezoidal space does not require as large number of pixels to be existent as that in the triangular space. Instead, the intermediate values in the trapezoidal space are determined by the four limiting vertices. However, the relatively more ground-based parameters in the *VI-T_s_* trapezoidal space than that in the triangular space have constrained the broad applications of the trapezoidal space. Some limitations have also emerged in *WDI* although this new index offers large opportunity than CWSI [120], including that: 1) there are no consideration of heat exchanges between soil and vegetation, which may be not valid when soil and vegetation are at different temperatures; 2) water stress does not have instantaneous effect on vegetation cover; 3) *WDI* method does not separate plant transpiration from soil evaporation.

#### 2.2.2. Dual-Source Model (Also Called Two-Source Model)

Although single-source energy balance models may provide reliable estimates of turbulent heat fluxes, they often need field calibration and hence may be unable to be applied over a diverse range of surface conditions. Kustas *et al.* [55] have shown that single-source models had serious limitations over partially vegetative surfaces, though some adjustments to *r_a_* can be made, but such adjustments are not generally applicable to all circumstances. Errors in sensor calibration, atmospheric corrections, and the specification of the surface emissivity have been detrimental to methods that rely on absolute surface temperature or surface-air temperature difference to derive regional surface energy balance [137]. Furthermore, air temperature measured at a shelter-level as an upper boundary condition suffers significantly from the interpolations over large heterogeneous areas [137]. Dual-source models require no a priori calibration and do not need additional ground-based information as that required in a single-source model and therefore have a wider range of applicability without resorting to any additional input data. Anderson *et al.* [38] showed that dual-source models represented an advance over single-source surface models that treated the earth's surface as a single, uniform layer. However, assumptions on and solution of dual-source energy balance models generally involve an estimation of the divergence of surface energy balance inside the canopy and the way to account for the clumped vegetation, which affects both the wind speed profile and radiation penetration and radiative surface temperature partitioning between soil and vegetation [42].

Generally speaking, the solution of a dual-source energy balance model is to implement the decomposition of the soil and canopy component temperatures either by iterating latent heat fluxes with the assumption that the vegetation is unstressed and transpiring at the potential rate or by acquiring remote sensing data of surface temperatures at multiple angles for the calculation of the component energy balance of soil and vegetation respectively.

The ensemble directional radiometric surface temperature [*T_RAD_*(*θ*)] is determined by the respective fraction of soil and vegetation viewed by a radiometer, which can be expressed as:

(30) $$T_{\text{RAD}}(\theta )={\left[f(\theta ){\text{T}}_{c}^{M}+(1-f(\theta ))T_{0}^{M}\right]}^{1/M}$$

where *M* is usually set to 4 for 8-14 μm and 10-12 μm wavelength bands.

If the surface emissivity and sky conditions are known, the directional radiometric temperature can be calculated from the brightness temperature [*T_B_*(*θ*)] from the following formula:

(31) $$T_{B}(\theta )={\left[\varepsilon (\theta ){\left(T_{\text{RAD}}(\theta )\right)}^{M}+(1-\varepsilon (\theta )){T_{\text{SKY}}}^{M}\right]}^{1/M}$$

With the assumption that the flux of soil surface is in parallel with the flux of leaves of canopy, and with a first-guess estimate of canopy transpiration (*LE_c_*) using Priestly-Taylor equation, which often leads an over-prediction in semiarid and arid ecosystems, *H* in a two source model can be divided into two parts of energy component of soil and vegetation:

(32) $$H=\rho c_{p}\frac{T_{\text{RAD}}(\theta )-T_{a}}{r_{rc}}=H_{s}+H_{c}=\rho c_{p}(\frac{T_{0}-T_{a}}{r_{a}+r_{s}}+\frac{T_{c}-T_{a}}{r_{a}})$$

Inputs to dual-source energy balance models generally include directional brightness temperature, viewing angle, fractional vegetation cover or leaf area index, vegetation height and approximate leaf size, net radiation, air temperature and wind speed. If measurements of *T_a_, u*, measurement heights, *T_RAD_*(*θ*) measured simultaneously at two viewing angles (e.g., data available from ATSR), canopy height (*h*), approximate leaf size, and fraction of vegetative cover (*F_c_*) or *LAI* are given, *T_c_, T_0_, H_c_, H_s_, LE_c_* and *LE_s_* can then be solved directly with the dual-source surface energy balance models without resorting to empirically determined ‘adjustment’ factors for “excess” resistance [40-41].

A series of papers have concentrated on the respective temperature and radiation components of both soil and vegetation through a set of applications, validations and modifications to the dual-source energy balance models over various landscapes over the past years [37,40-41,138-149]. The increase of surface temperature in the morning was also found to be highly sensitive to the change of surface soil moisture (and thus ET) [9,19,26,67,150-152] and an utilization of rate of surface temperature rise in the form of simplified equation has also been shown by Carlson and Buffum [26] to estimate daily ET with the advantages of no need for absolute surface temperature retrievals from satellite data. Wetzel *et al.* [150] and Diak [151] have attempted to compute surface energy balance by using the rate of rise of *T_s_* from a geostationary satellite with an atmospheric boundary layer model. Norman *et al.* [37] developed a TSM (Two-Source (soil+canopy) Model) to accommodate the difference between radiometric surface and aerodynamic temperatures to partition surface energy balance into energy components of both soil and vegetation using data either from a single view angle or from multiple view angles. Subsequently, on the basis of that work, Anderson *et al.* [38] examined and tested the TSTIM (Two-Source Time Integrated Model, subsequently was named as ALEXI: Atmosphere-Land Exchange Inverse [137]) relating the morning rise of surface temperature acquired at 1.5 and 5.5 hours past sunrise to the growth of a planetary boundary layer through an estimate of sensible heat using data collected during ISLSCP and Monsoon '90 experiments. Lhomme and Chehbouni [153] have commented on the assumption on the parallel transfer of heat from canopy and soil and assumed the scale to be a determinant of whether a dual-source model should be coupled or not. Since 1999, ALEXI has been applicable over a wide variety of landscape, agricultural and land-surface-atmosphere interactions [146]. It removes the need for the measurements of near-surface air temperature and is relatively insensitive to uncertainties in surface thermal emissivity and atmospheric corrections on the remotely sensed surface temperatures. Kustas and Norman [42] made four modifications, which had largest impacts on dual-source flux predictions under sparse canopy-covered conditions to the TSM developed by Norman *et al.* [37], involving: 1) the estimation of the divergence of net radiation with a more physically-based algorithm; 2) use of a simple model to account for the effects of clumped vegetation; 3) application of an adjusted Priestley-Taylor [154] coefficient; 4) computation of soil resistance to sensible heat flux transfer with a new formulation. Norman *et al.* [143] developed a variation of TSM called DTD (Dual-temperature-difference) method using time rate of change in *T_s_* and Ta to derive surface turbulent fluxes and this DTD method is simpler than other modifications of TSM in that it requires minimal ground-based data and does not require modeling boundary layer development. On the basis of TSTIM, a two-step approach called DISALEXI (Disaggregated ALEXI) model has been proposed to estimate surface ET with the combination of low- and high-resolution remotely sensed data without a need for local observations [143, 155]. Anderson *et al.* [147] have found that consideration of vegetation clumping within the thermal model could significantly improve the estimates of turbulent heat fluxes at both local and watershed scales when observations from eddy covariance data collected by aircraft and a ground-based tower network are compared. Li *et al.* [148] compared two resistance network formulations that are used in a dual-source model for parameterizing soil and canopy energy exchanges over a wide range of soybean and corn crop cover and soil moisture conditions during the Soil Moisture-Atmosphere Coupling Experiment. In the two resistance formulations, the parallel resistance formulation does not consider interaction between the soil and canopy fluxes while the series resistance algorithms provide interaction via the computation of a within-air canopy temperature. Results from Li *et al.* [148] showed that both the parallel and series resistance formulations produced basically similar estimates compared with the tower-based flux observations while the parallel resistance formulation was more able to achieve the balance of the component temperature and heat fluxes of soil and canopy. Sanchez *et al.* [139] applied a simplified two-source energy balance model by using a “patch” treatment of the surface flux sources to predict the partitioning of net radiation into the components of soil and vegetation over a maize crop in Beltsville MD, USA during the 2004 summer season. Anderson *et al.* [156] developed a multiscale Land-Atmosphere Transfer Scheme based on the ALEXI and DISALEXI models to upscale tower and air craft data to larger scales with inputs mainly being surface temperature and vegetation cover. Li *et al.* [157] tested and compared respectively the utilities of both microwave-derived near surface soil moisture and thermal-infrared surface temperature in a two-source energy balance model and model performance under those two cases was assessed by comparing with data collected from a network of 12 METFLUX towers. Promising results with flux values agreeing within 50 W/m^2^ have also been obtained by comparison of flux calculated respectively from a two-source energy balance model and SEBAL with ground based measurements acquired over an experimental site in central Iowa, USA [158].

Compared to other types of remote sensing ET formulations, dual-source energy balance models have been shown to be robust for a wide range of landscape and hydro-meteorological conditions [40]. The ALEXI approach is believed to be a practical means to operational estimates of surface fluxes over continental scales with the spatial resolution of 5- to 10-km.

The main advantages of the dual-source models over single-source models are that: 1) they avoid the need for precise atmospheric corrections, emissivity estimations and high accuracy in sensor calibration; 2) ground-based measurement of *T_a_* is not indispensable when dual-source models are coupled with a PBL [13] and thus is much better suitable to applications over large-scale regions than single-source models and other algorithms [38]; 3) they generally incorporate effects of view geometry; 4) they avoid empirical corrections for the ‘excess resistance’. However, applications of the aforementioned models of both directly relating surface turbulent fluxes to temperature difference measured at two times and imbedding the morning temperature rise into a dual-source energy balance coupled with a PBL (Planetary Boundary Layer) generally require data from a geo-stationary satellite, which is less suitable for high latitudes due to the suboptimal viewing orientation and coarse spatial resolution to provide a series of cloud-free images [83]. The new MSG/SEVIRI (Meteosat Second Generation/ Spinning Enhanced Visible and Infrared Imager) sensor has provided a good promise with its relatively small pixel size and high observation frequency for applications in Europe and Africa.

### 2.3. Data Assimilation

Results from remote sensing ET models are generally either instantaneous (daily) values using data from polar-orbiting satellites or coarse spatial resolution values from geostationary satellites, which can not provide temporally continuous values and thus cannot meet the requirements of most hydrological and numerical prediction models. One possible means to overcome this dilemma is to use data assimilation techniques to map ET, which can take advantage of the synergy of multisensor/multiplatform observations [35,159].

Data assimilation has been firstly used by meteorologists to construct daily weather maps, displaying variations of environmental variables such as pressure and wind velocity over space and time [160]. Simply speaking, data assimilation technique is the process in which all available information is used in order to estimate objective variables as accurately as possible [161-162]. A data assimilation system is generally consisted of three components: a set of observations, a dynamic model and a data assimilation technique [163]. All existing assimilation algorithms can be described as more or less an approximate of statistical linear estimation [164]. Data assimilation schemes are often statistically optimal by minimizing the errors in estimates derived from merging noisy observations and uncertainty of models in a statistical sense.

Data assimilation techniques for ET estimates can assimilate all available information but they generally have to rely on a numerical model which may need a lot of atmospheric forcing and is relatively computationally demanding than remote sensing ET models [165-166]. Selection of a data assimilation technique is essentially to achieve a balance between making the best use of all available information (optimality) and computational efficiency, flexibility, and robustness. However, compromises have to be made to adapt to specific goals because these evaluation criteria often conflict [167]. The principle of any data assimilation scheme is to minimize the mismatch between the observations and models by adjusting components under the fundamental physical constraints.

Nowadays, data assimilation techniques are generally put into two categories, including sequential assimilation (e.g., Ensemble Kalman Filter and optimal interpolation) [168-174] and un-sequential/variational/retrospective assimilation (e.g., 4-dimentional variational assimilation) [175-179]. One of the distinctions between sequential and variational assimilations is that in sequential assimilation each individual observation influences the estimated state of the flow only at later times and not at previous times while variational assimilation aims at adjusting the model solution globally to all the observations available over the assimilation period [161]. Several papers have attempted to use data assimilation techniques combined with a numerical model to estimate regional surface turbulent heat fluxes [35,180-183]. Boni *et al.* [35] developed a land data assimilation system to estimate latent heat flux and surface control on evaporation with the dynamic equations for surface temperature as the constraint. In this assimilation system, satellite remotely sensed surface temperatures are assimilated within the Southern Great Plain 1997 hydrology field experiment. Factors characterizing land surface influences on evaporation and surface heat fluxes are estimated through assimilation of radiometric surface temperature sequences with a land surface energy balance as a constraint and this approach has been tested using data from the ISLSCP FIFE (International Satellite Land Surface Climatology Project) [181]. Caparrini *et al.* [184] proposed a land data assimilation scheme with sequences of multi-satellite remotely sensed surface temperature measurements and data from surface micrometeorological stations to estimate the surface energy balance components in a basin with varying surface conditions. Margulis *et al.* [182] compared the *VI-T_s_* triangle method to variational data assimilation method for estimating surface turbulent fluxes from radiometric surface temperature observations. Results from a set of synthetic experiments and an application of data from ISLSCP FIFE site have shown that the assimilation approach performs slightly better than the *VI-T_s_* triangle method.

The data assimilation approach to mapping surface energy fluxes often has some advantages over traditional retrieval methods, including: 1) assimilation procedure estimates not only latent heat flux but also the various intermediate variables related to the turbulent heat fluxes in a numerical model; 2) estimates of the turbulent heat fluxes are continuous in time and space since the dynamic models used in the assimilation procedure interpolate the measurements taken at discrete sampling times; 3) the data assimilation procedure can produce estimates at a much finer resolution; 4) data assimilation scheme can merge spatially distributed information obtained from many data sources with different resolutions, coverage, and uncertainties [167]. The main drawback of data assimilation techniques to retrieve regional ET with a numerical model is that they are relatively more computationally demanding than the remote sensing ET models.

Above mentioned sub-sections detail the theory, advantages and weaknesses of the various remote sensing ET models from the simplified empirical regression method applied over a field scale to the relatively complex dual-source surface energy balance models employed at both regional and continental scales. Data assimilation approaches can assimilate all available data sources to provide the spatially and temporally continuous surface turbulent heat fluxes. Comparisons of the different remote sensing ET models reviewed above are recapitulated in Table 1.

## 3. Scaling from Instantaneous ET to Daytime Integrated Value

### 3.1. Sine Function

Most of the aforementioned ET models using remotely sensed data produce only instantaneous ET values. Obviously, it is necessary to convert essentially instantaneous ET value at the overpass times of satellites to daily or longer time value to make full use of the remote sensing data in hydrological and water resources management applications. A number of techniques are proposed to extrapolate the instantaneous ET to the longer time values, either through use of a numerical model that simulates the diurnal surface temperature under realistic air temperature, wind speed and humidity conditions or by giving some assumptions on the relationship between instantaneous ET (*ET_i_*) and daily values (*ET_d_*). Jackson *et al.* [62] related the ratio of instantaneous ET to daily value to the diurnal trend of solar irradiance with the following equation:

(33) $$ET_{d}/ET_{i}=R_{sd}/R_{si}=2N/(\pi \sin(\pi t/N))$$

where subscripts *d* and *i* respectively indicate the daily total and instantaneous values. The sine function gives a good approximate of the change of diurnal solar irradiance except near sunrise and sunset. *N* is the duration of daytime and can be expressed as:

(34) $$N=0.945(a+b\sin^{2}(\pi (D+10)/365))$$

(35) $$a=12.0-5.69\times 10^{-2}\lambda -2.02\times 10^{-4}\lambda^{2}+8.25\times 10^{-6}\lambda^{3}-3.15\times 10^{-7}\lambda^{4}$$

(36) $$b=0.123\lambda -3.10\times 10^{-4}\lambda^{2}+8.00\times 10^{-7}\lambda^{3}+4.99\times 10^{-7}\lambda^{4}$$

With Equations (33-36) and time of day (*t*), day of year (*D*) and geographical latitude *Λ*between 60° S and 60° N, one can scale the one-time measured instantaneous ET to the daily totals. Jackson *et al.* [22] have shown that when the daytime was always cloud free or the cloud cover was relatively constant throughout the daytime, the sine function of Equation (33) could obtain reliable estimates of daytime integrated ET. When cloudy days exist, improvements of Equation (33) should be made to take account the mount and temporal coverage of the cloud cover. This approach is widely used for daily ET estimation and satisfactory results have been produced [13,39,185,186]. Zhang *et al.* [185] refined the sine function by introducing a parameter to reflect impacts of geographic latitude, solar declination and degree of cloudiness on the convexity of the diurnal patterns of solar radiation.

### 3.2. Constant Evaporative Fraction (EF)

Sugita and Brutsaert [187] assumed the evaporative fraction to be constant during the daylight hours to determine regional daily ET using data obtained during FIFE in northeastern Kansas. Knowing the daytime available energy (*R_n_-G*)*_d_*, and assuming that EF is constant during the daytime, daily estimate of *ET_d_* can therefore be written as:

(37) $$LE_{d}={\left(R_{n}-G\right)}_{d}\cdot EF_{i}={\left(R_{n}-G\right)}_{d}\frac{LE_{i}}{{\left(R_{n}-G\right)}_{i}},$$

where subscripts *i* and *d* are respectively indicative of instantaneous and daytime integrated values. The value of EF varies from 0 to 1 under daytime convective conditions with minimal advection and represents the fraction of available energy partitioned into latent heat flux [188].

A great number of papers have used this assumption to calculate the daily ET and examined whether the assumption that daytime EF is nearly constant throughout the day is reasonable [186-195]. With data from the FIFE and other observations, Crago [190] has concluded the variability or conservation of EF on individual day was affected by complicated combination factors, including weather conditions, soil moisture, topography, biophysical conditions, cloudiness and the advections of moisture and temperature directly contributed to the amount of variability of EF on a given day. A strong correlation with the coefficient of determination value of 0.89 has been demonstrated between the midday and daily average evaporative fractions for data from the Hapex-Mobilhy program on clear days [191]. Zhang and Lemeur [185] using data from the Hapex-Mobilhy Experiment in southwestern France compared the sine function with the constant EF method and concluded that both methods were accurate to estimate daily total ET for cloud-free days and recommended that the sine function was preferable for the purpose of estimating ET using remotely sensed data.

Jackson *et al.* [62], Kustas *et al.* [23], and Owe and van de Griend [196] have found that nighttime ET could reach as many as about 10 percent of the daily totals. Allen *et al.* [36] illustrated that the assumption of constant EF during the 24 h period could underestimate the overall daily ET when afternoon advection and increased wind speed appeared in arid climates. Anderson *et al.* [38] therefore added this 10 percent of latent heat fluxes into daily integrated ET in Equation (37) using the evaporative fraction expressed as follows:

(38) $$EF=1.1\frac{LE_{i}}{{\left(R_{n}-G\right)}_{i}}$$

### 3.3. Constant Reference ET Fraction (ETrF)

In the METRIC process, Allen *et al.* [36] proposed a constant *ET_r_F*, which is believed to be better able to capture any impacts of advection and changing wind and humidity conditions during the day, to estimate the 24 h total ET. *ET_r_F* is defined as the ratio of the computed *ET_i_* from each pixel to *ET_r_*. *ET_r_*. is the reference ET over the standardized 0.5 m tall alfalfa and computed from meteorological data measured at ground meteorological stations [36]:

(39) $$ET_{r}F=\frac{ET_{i}}{ET_{r}}$$

(40) $$ET_{i}=3600\frac{LE}{L\times \rho_{w}}$$

(41) $$L=[2.501-0.00236(T_{s}-273.15)]\times 10^{6}$$

With the assumption of instant *ET_r_F* being same as the average *ET_r_F* over the 24 h average and the consideration of the sloping effects over terrain areas, *ET_d_* can be estimated by [36]:

(42) $$ET_{d}=C_{\text{rad}}(ET_{r}F)(ET_{r,d})$$

(43) $$C_{\text{rad}}=\frac{R_{s,i,\text{Horizontal}}}{R_{s,i,\text{pixel}}}\cdot \frac{R_{s,d,\text{pixel}}}{R_{s,d,\text{Horizontal}}}$$

where subscripts *i* and *d* indicate instantaneous and daily values respectively; subscripts “pixel” and “horizontal” represent respectively the value for a specific pixel at certain slope and aspect conditions and value calculated for a horizontal surface. For applications to horizontal areas, *C_rad_* = 1.0. *ET_r,d_* is cumulative daily reference ET [36].

## 4. Problems/Issues

Although great progress has been made since 1970s on a number of methods from an empirical simplified equation to a more complex physically based dual-source energy balance model using the remote sensing technology to estimate regional surface turbulent fluxes, there are still some problems that have not been solved reasonably, which are mainly associated with the parameterization of land surface fluxes at regional/global scales, retrieval accuracy and physical interpretation of different surface variables retrieved from satellite data, temporal and spatial data/model scaling from one scale to other scale, validation of the latent heat flux obtained from models at regional/global scale, etc. These problems will be discussed briefly below.

### 4.1. Problems Related to Remotely Sensed Data Itself

Remotely sensed data are acquired instantaneously and can only provide instantaneous two-dimensional spatial distribution of land surface variables such as surface albedo, surface vegetation fraction, surface temperature, surface net radiation and soil moisture, etc., which are indispensable variables to know for remote sensing estimate of land surface ET. This is one of the specialities of remote sensing technique, as well as the distinct predominance of remote sensing technique in estimating spatial distribution of land surface ET at regional/global scale. These speciality and predominance have great impact on the spatial scaling from the “point” to the regional scale. However, temporally integrated daily, weekly and monthly ETs at regional and global scales are required for many ET-related disciplines. Therefore, temporal scaling, which is one of the weaknesses of remotely sensed data, is needed to convert the instantaneously spatial ET to a longer-time value. Moreover, due to the effect of cloud coverage, it is impossible to provide the two-dimensional spatial patterns of land surface variables under the clouds by the optical remote sensing and consequently impossible to estimate the surface instantaneous ET over the areas covered by clouds with optical remote sensing data. Nowadays, great progress has been made to convert the instantaneous ET to the daily value on clear-sky days while little work or progress has been done on the temporal scaling from instantaneous remote sensing ET to weekly/monthly remotely based-ET due to the coarse spatial resolution of microwave remote sensing data and the inaccuracy of the surface variables used in remote sensing models retrieved from the microwave data, as well as the effects of cloud cover.

### 4.2. Uncertainty of the Remote Sensing ET Models

Over the past 30 years, a variety of remote sensing ET models have been developed to estimate the spatial distribution of ET at various scales ranging from the field (simplified empirical equation) to regional (single-source or dual-source models) and continental scales (eg. ALEXI). Single-source models can be applied with a relatively high accuracy over homogeneous areas (eg. dense vegetation), while over the arid and semi-arid areas (eg. partially vegetated cover) two-source models are especially required to separately model the heat interactions between soil and atmosphere and between vegetation and atmosphere. However, as reviewed in the previous sections, each model developed has its advantages and disadvantages (weaknesses) and was applied successfully to some extent to some conditions. Since, in the different areas of the world, there exist great differences in the land surface characteristics, in the climate and terrain etc., no model developed nowadays can be used everywhere in the world without any modification or improvement to estimate the ET from satellite data. A big challenge in the development of remote sensing ET model is to develop a new parameterization of land surface ET with only land surface variables and parameters directly or indirectly derived from satellite data.

### 4.3. Uncertainties in the Accuracy of the Retrieved Land Surface Variables (Parameters)

The presence of the atmosphere between land surface and sensors at satellite level disturbs the radiances measured by a radiometer at the top of the atmosphere. These radiances result primarily from emission/reflection of surface modulated by the effects of absorption, diffusion and emission of the atmosphere. The passage of the radiances measured at the top of the atmosphere to the macroscopic land surface parameters (variables) and physics of surfaces requires the corrections for the atmospheric effects and the connection of the surface parameters (variables) derived directly from satellite data to other surface parameters (variables) through physical models.

Although great progress has been made nowadays to retrieve quantitatively land surface variables (parameters) from remotely sensed data, accuracy of some variables (parameters), such as surface temperature, *LAI*, vegetative coverage, plant height, etc., required in remote sensing ET models still needs to be improved. In addition, due to the influences of vegetation architecture, sunlit fractional of vegetation and solar zenith angle, etc., observational angular effect is a significant factor affecting the retrieval of radiometric surface temperature especially over heterogeneous surfaces [37]. Differences in received radiances will occur due to the differing amounts of soil and vegetation in the filed of view when sensor viewing changes from one angle to another, while over homogeneous dense, well-watered vegetative surfaces, the effect is less important [25,38]. Data obtained during the ISLSCP FIFE program have shown that difference of surface temperature obtained at nadir and 60 degrees in zenith angle can reach as large as 5 °C [38], implying that a large and unaccepted error on ET estimate would be generated if the angular effect is neglected. In order to take into account this angular effect in the development of dual-source remote sensing ET models, methodologies must be developed to estimate accurately the component temperatures of surface (vegetation and ground) from multispectral and multi-angular satellite measurements.

### 4.4. Lack of the Measurements of Near-Surface Meteorological Variables

In most remote sensing data based ET models, whatever the single-source or dual-source models are, meteorological data (air temperature, atmospheric pressure, wind speed, relative humidity) at PBL-height or at near-surface height at satellite pixel scale are indispensable and spatial interpolation method is often used to get these meteorological data at satellite pixel scale from discrete meteorological stations. Because the big difference of the climate and terrain conditions may exist in the study region and the implementation of meteorological stations is often sparse and irregular in the world, accuracy of the non-physics and merely spatial-statistics based interpolation needs to be improved either by developing physically static- or dynamic-feedback interpolation methods based on remote sensing data or by making use of atmospheric reanalysis data at high spatial-resolution. Another approach to improve the accuracy of spatial data interpolation is to integrate the remote sensing ET models with atmospheric general circulation models or numerical weather forecast models, which maybe one of the promising subjects in the future for the regional ET estimates with remotely sensed data.

### 4.5. Spatial and Temporal Scaling Effects

The scaling problem is of a much more fundamental nature since it implies a conceptual analysis of the physical significance of the measured quantities (variables). Indeed, the diversity of continental surfaces involves spatial (vertical and horizontal) and radiometric heterogeneities of surface, considering the spatial resolution of the current onboard sensors varying from 10^-2^ to 10^1^ km^2^, it is therefore necessary to be able to define and interpret correctly surface parameters (variables) independent of the scale used, as well as the processes necessary to validate this definition.

Simply speaking, scaling effects in the derivation of surface turbulent fluxes are shown in the form of whether functions of parameters and variables obtained over one scale can be used at other scales (local/regional/large) [25]. It seems general that models applicable for deriving surface fluxes/parameters at local scale may not be appropriate for applications at a larger scale because of the heterogeneities of the surface and non-linearity of the models [25].

Since 1980s, several international field programs have been designed to obtain useful surface parameters and study the issue of scaling from point to regional- or global-scale estimates of the surface energy fluxes [25]. The spatial resolution of thermal infrared bands is usually coarser than that in visible and near infrared bands, which will lead to a scale difference in the land surface parameters indispensable to ET estimates between surface temperature obtained from thermal bands and vegetation indices derived from visible and near infrared bands [24, 82].

The possibility of resolving all problems raised by scaling effects may be to a great extent associated with the development of the scaling theory and further with the fusion of multi-scale remote sensing observations [14,82].

### 4.6. Lack of the Land Surface ET at Satellite Pixel Scale for the Truth Validation

Comparisons between turbulent heat fluxes derived from remote sensing ET models and in-situ measured data are required to evaluate the reliability and accuracy of the applied ET models. Although it may be feasible and reasonable to validate pixel-averaged fluxes derived from remote sensing ET models with traditional measurements mainly conducted at the “point” scale over uniform areas, problems will be encountered when validation is performed over complicated land surface areas.

Nowadays, several conventional techniques such as Bowen ratio, eddy correlation system and weighing lysimeters have been commonly applied to measure the ET at ground level. Lysimeters provide the only direct measure of water flux from a vegetated surface. Its measurements can therefore be used as a standard for evaluating the performance of other physically based ET models. However, data measured by Lysimeters are essentially point data and thus cannot be used for validating the regional ET estimates [28]. Study has shown that measurements from Bowen ratio and large weighing lysimeters for irrigated alfalfa during advective conditions can differ by up to 29% [197-198]. Eddy correlation technique, based on the principle that atmospheric eddies transport the entities of water vapor, CO_2_, and heat with equal facility, is particularly useful for rough surfaces with high coefficients of turbulent exchange [28]. It has overtaken Bowen ratio as being the most preferred micrometeorological technique for ET measurements in the past few decades [199]. The source area of an eddy correlation system generally represents an upwind distance of about 100 times the sensor height above the surface [200], which is appropriate to validate the ET at pixel sizes of an order of hundred meters. In the past decades, most studies used measurements conducted by the Bowen Ratio Energy Balance (BREB) and the eddy correlation system to validate ET at local and regional scales. Angus and Watts [201] showed that LE measured by Bowen ratio was dependent on the range of Bowen ratio values. For ET at the potential rate, relative errors of up to 30% in Bowen ratio can produce relative errors of 5% in LE. However, as soil water becomes less available, the precision in LE will decrease [78]. Energy balance non-closure in eddy correlation, typically higher over strongly evaporating surfaces such as irrigated crops [199], can reach up to 20%, even for non-advective conditions [82]. Measurements from eddy correlation system at night under low wind-speed stable conditions can yield large errors and the instrument errors and atmospheric stability contribute to the sources of errors [69,202].

Validation of remote sensing ET derived from satellite data at high spatial resolution, such as TM and ASTER data, was generally performed using the measurements made by the BREB and eddy correlation system. However, difficulties still remains in validation of ET estimated from low spatial resolution satellite data such as MODIS, GOES whose pixel size in thermal bands is a magnitude of an order of kilometers [25].

The newly developed (Extra-) Large Aperture Scintillometers (XLAS, LAS) provide a promising approach to validate the remote sensing ET at much larger scales [203-206]. Scintillometers are regarded as the unique possibility of measuring the sensible heat flux averaged over horizontal distances comparable to the grid size of numerical models and satellite images [207] and thus can be employed to validate to a certain degree the regional turbulent heat fluxes derived from remote sensing models. One limitation of using Scintillometers is the saturation of scintillation, which can be overcome by using either large, incoherent transmitter and/or receiver apertures or a longer wavelength [207-208].

## 5. Future Trends and Prospects

From what was mentioned previously, if there are no innovated methods in acquisition of remotely sensed data and meteorological variables or newly-developed ET models, the main restricting factors in the estimates of actual instantaneous/daily/weekly/monthly ET over regional scale from remote sensing techniques are actually the retrieval accuracy and physical interpretation of different surface variables retrieved from satellite data, parameterization of land surface fluxes at regional scale, temporal and spatial data/model scaling among different scales, validation of the latent heat flux obtained from models at regional scale, acquisition of near-surface meteorological data over different satellite pixel scales etc.

As known, the sensors onboard satellites only measure radiances at the top of the atmosphere. These measured radiances are in general the quantities integrated over very heterogeneous and large surfaces. One can thus ask following questions: Can one extract from these radiances the macroscopic parameters (variables) describing such a surface? Do such macroscopic parameters exist? How to define them? One can also wonder whether the description of the physical processes at the land/atmosphere interface developed at local scale is applicable to the larger (spatial) scale with surface parameters (variables) integrated over this surface. The attempts to answer all these questions lead to study the fundamental and conceptual aspects of the definition of the macroscopic parameters (variables) and the scaling effects. The passage of the radiances measured at the top of the atmosphere to the macroscopic parameters (variables) and physics of surfaces requires the corrections for the atmospheric effects and the connection of the surface parameters (variables) derived directly from satellite data to other surface parameters (variables) through physical models. These problems lead to study the methodological aspects of the derivation of the surface parameters (variables) which can not be retrieved directly from satellite data and the metrology aspects of the atmospheric corrections necessary to the determination of other surface parameters (variables) directly from satellite data. Study focusing on the following subjects in the future will be recommended for quantifying regional and global ET.

### 5.1. Modeling of Land Surface Processes at Interface of Soil-Biosphere-Atmosphere at Regional Scale

This modeling aims to formulate the processes of exchanges between soil-biosphere-atmosphere in terms of macroscopic parameters which have significant physical meaning at regional scale and are measurable from satellites. The required formulation should permit to specify both the physical meaning of the measurements by satellite and the passage of local scale to regional and global scales. It concerns a semi-phenomenological analysis which could lead to a new method to assimilate effectively satellite data for the land surfaces.

#### 5.1.1. Dialectical Approach to Model the Spatial-Temporal Variations of Land Surface Processes at Various Scales

Two modeling methods (one is based on the other) can be developed to study what occurs at regional and global scales.

##### 5.1.1.1. Integrating Method

This method consists in describing all the elements that compose a pixel, in modeling the processes for each one of these elements and extrapolating these models by a process of “surface integration” to deduce what occurs to large scales. It is about a type of up-scaling. Because of the non-linearity of the processes, this integration is complex and is based on assumptions not always easy to control. This method is very useful to understand what occurs, and can direct the research of the “integrating” variables (parameters) directly describing the processes at the scale considered. It is however difficult with this method:

- to benefit from “simplifications” which must appear at large scale, due to the fact that one cannot measure all the characteristics of the elements composed the pixel.
- to highlight “the good” variables representative of the system at large scales.

This method can lead to models having a very great number of parameters and variables whose determination at large scales is not possible without arbitrary, taking into account the extreme local variability of these in-situ quantities.

##### 5.1.1.2. “Autonomous” Method at Large Scales

Although the integrating method is very rich and useful, it must be supplemented, in a dialectical way, by a method that analyzes and models (parameterizes) the observations made directly at satellite pixel scale. This second method is founded on the principle of “scale autonomy” which implies that the processes at a given scale can be described and understood at this scale in an autonomous way and without making reference to the phenomena and processes intervening at a lower scale, even if they are the consequence. The passage from one scale to others permits to describe the parameters and variables defined in a given scale in function of the variables and processes of under systems intervening on a lower scale.

This obviously raises the question about whether this autonomous description with large scales exists and whether necessary and sufficient measures are currently available to carry out this study. As only satellite measurements are available, the question is whether necessary and sufficient variables (parameters) can be defined with these satellite measurements to describe the state of surface and processes of the land surface at satellite pixel scale. The answer is not obvious [209] and is not really known. However, experiments showed that it is possible to highlight spatial indicators which are sensitive to the variations of the state of the biosphere [210-213]. It is not possible to currently give an exhaustive list of these indicators. One can nevertheless quote a certain number of it: surface temperature, temporal sums of temperature, vegetation indices, Microwave Polarization Difference Indices (MPDI), complex inertia, albedo, precipitation indices, moisture indices, roughness indices, resistance indices and temporal sums of some of these indices, etc.

It was shown that these indices are not independent and it is possible to establish laws between their variations. Indeed, recent studies seem to indicate that this method is possible and can constitute an original approach of the processes at regional scale or global scale without going into the details of the local scale. For example, it was shown that relations NDVI/Ts could be correlated with evapotranspiration resistance, with surface moisture [112,113,214,215] and that the correlations albedo/*T_s_* could provide an indicator permitting to monitor extension of the area affected by the desertification [216,217]. It was also showed that the correlations between visible reflectances and MPDI could characterize the interannual variations of the soil surface due to the hydrous deficit [218,219]. They are yet only the preliminary studies, but they indicate nevertheless potential and very interesting research for such an “autonomous” approach.

Although still very little developed, such an “autonomous” approach is now feasible. Indeed, huge space measurements provided by the earth observation satellites are now available to scientists and they can now begin to be able to derive an ensemble of important surface variables (parameters) and/or spatial indicators from these measurements.

#### 5.1.2. Reformulation of the Energy Balance at Large Scales

Efforts will be made by introducing “integrating parameters” and a parameterization of the diurnal variation of surface temperature with a minimum number of parameters into the reformulation of the energy balance at large scales [220] in order to use the temporal information provided by satellite data. To simulate complicated phenomena, one can try to introduce measurable parameters from space, such as for example a complex inertia or complex coefficients of transfer [221,222].

#### 5.1.3. Phenomenological Analysis of the Spatial-Temporal Variations of the Spatial Indicators Characterizing Surface States and Processes at Satellite Pixel Scale

The phenomenological analysis suggested will be carried out to allow:

- description of phenomenological relations between surface variables and/or spatial indicators and to reveal possibly new parameters characterizing land surface states and processes, to highlight characteristic thresholds of the release of certain phenomena (erosion, release of sandstorm, degradation etc…),
- establishment of the laws and properties which take into account these variations,
- to study these laws and the stability of the processes which they describe in function of the parameters controlling these laws.

According to the above cases, this analysis could be performed using the signal processing methods for nonlinear systems, and one will focus to study the way in which the variations observed and modeled change in function of the value of the parameters controlling the equations which will be established.

#### 5.1.4. Modeling and Assimilation of the Data

A big challenge in the development of remote sensing ET models is to develop a new fully remote sensing data-based parameterization of land surface ET with only land surface variables and parameters directly or indirectly derived from satellite data.

Associating the measurements taken from satellites with land surface models is essential to connect between measurements and models, the various surface characteristic state variables (parameters) (or other relevant parameters), the parameters of process, and the space observations. Efforts will be made to introduce modifications of the existed land surface models by assimilating satellite data and if possible by introducing a new parameterization of land surface evapotranspiration process and evaporative fraction based on the relevant parameters observed from space. This aspect is very important to correctly take into account the effects of feedback. Accordingly, it will undoubtedly be necessary to reformulate certain equations to introduce parameters directly accessible to space measurement, or to re-compute these parameters from the models.

### 5.2. Further Improvement of the Accuracy of Land surface Variables (Parameters) Retrieved from Remotely Sensed Data

Land surface temperature is the direct indicator of how much energy and water could be available over the land surface and is one of the most key factors affecting the accuracy of the ET estimates. Land surface temperature along with other related remotely sensed surface variable (parameters) such as surface albedo, emissivity, *NDVI*, soil moisture, fractional vegetation cover and *LAI* in the energy balance models have significant impact on the precise partition of the four energy components in these models and consequently on the accuracy of the retrieved regional ET. Although great progress has been made nowadays to retrieve quantitatively land surface variables (parameters) from remotely sensed data, accuracy of some surface variables (parameters) required in remote sensing ET models still needs to be improved and more attention should be paid also to the physical interpretation of these surface variables (parameters) retrieved directly or indirectly from satellite data.

### 5.3. Research In-Depth on the Impact of the Advection on Regional Estimates of ET

Advection is another factor influencing the accuracy of the partition of surface available energy into turbulent fluxes. It often occurs in the urban area and desert and directly causes the imbalance of surface energy especially over small spatial scales (high spatial-resolution) and is another source of energy to evaporate the water from surface. At present, it is still uncertain over what scale advection will have to be considered and how the energy is exchanged between neighboring pixels in the horizontal direction.

### 5.4. Calibration of Land Surface Process Models with the Remote Sensing ET to Map Regional and Time-Integrated ET

Theoretically, remote sensing ET models can provide relatively accurate spatial distributions of instantaneous ET while land surface process models driven by atmospheric forcing data, and run with related surface data and physical properties of soil and vegetation as model inputs, can simulate the long-term development trend of the turbulent heat fluxes, soil water content and other related corresponding physical, chemical and biological processes that might occur over both temporal and spatial scales. Land surface process models may help to overcome the limitation of the current remote sensing ET models, the latter is merely employed under clear sky conditions and at instantaneous scale. However, because of both the low spatial resolution and the uncertainties in the model inputs in the land surface process models, it is hard, sometimes impossible, to estimate correctly the latent heat flux at a large scale with land surface process models without adding information provided by satellite data. Remote sensing has the unique advantages on the acquisition of spatial land surface variables (parameters) needed in the land surface process models from a scale of several meters to a scale of several kilometers. It may be an effective way to reduce the uncertainties existing in the current land surface process models. Efforts will be made therefore to develop methodologies to calibrate the ET simulated by land surface models with remote sensing ET values and use as many as possible all the land surface variables (parameters) derived from satellite data under clear sky conditions. In addition, with the rapid development of multi-spectral, multi-temporal and multi-spatial satellite technology, computer processing technique and optimization algorithms in the geosciences, data assimilation is believed to be another promising way to integrate the models, data and optimization methods together to estimate temporal and spatial ET continuously.

### 5.5. Validation of the ET and Land Surface Variables (Parameters) at Satellite Pixel Scale

Validation is the process of assessing by independent means the uncertainty of the data products derived from the system outputs. Without validation, any methods, models, algorithms, and parameters derived from remotely sensed data can not be used in confident. Both the fundamental physical measurements made by the sensor (e.g. radiance) and the derived geophysical variables (e.g. biomass) must be properly validated. Validation is the most key and urgent issue to be dealt with because validation can help to understand the combined effects of errors in the remotely sensed data, uncertainty in the remote sensing ET models and uncertainty in the retrieved land surface variables (parameters), and thus can provide feedback and some clues to optimize models, improve accuracies of both the remotely sensed data and the retrieved land surface physical variables (parameters).

Currently, validation of estimated ET is one of the most troublesome problems, mainly because of both the scaling effects, i.e., comparisons between remote sensing ET and ground-based ET measurements, and the advection effects. Several validation techniques have to be developed. These may include comparisons of remote sensing ET with ground-based ET measurements conducted over validation test sites, inter-comparisons with ET estimated from satellite data at different spatial resolution or estimated using combined various data sources and land surface process models, inter-comparison of trends derived from independently obtained reference data and remotely sensed data, and analysis of process model results which are driven or constrained by remotely sensed data and ET. However, due to the surface heterogeneity and scaling effects, it may be questionable to validate the turbulent heat fluxes at satellite pixel scale with the “point” scale measurements obtained from the Bowen ratio, lysimeter and eddy correlation system over non-uniform and heterogeneous surfaces. The newly developed LAS (XLAS) can provide a promising approach to validate the remote sensing ET at much larger scales.

## Figures and Tables

**Table 1.** Comparisons of a variety of commonly applied remote sensing ET methods.

**Methods**	**Refs.**	**Equations**	**Main inputs**	**Main assumptions**	**Advantages**	**Disadvantages**
Simplified Equation	[20] [22]	(1)	*R_nd_, T_s_, T_a_*	1) Daily soil heat flux is negligible; 2) Instantaneous H at midday can express the influence of partitioning daily available energy into turbulent fluxes.	Simplicity	Site-specific
*VI-T_s_* Triangle	[115]	(28)	*R_n_, G, T_s_, VI*	1) Complete range of both soil moisture and vegetation coverage exists within the study area at satellite pixel scale; 2) Cloud contaminations are discarded and atmospheric effects are removed; 3) *EF* varies linearly with *T_s_* for a given *VI*	No ground-based measurements are needed	1) Difficult to determine the dry and wet edges; 2) *VI-T_s_* triangle form is not easy recognized with coarse spatial resolution data
*VI-T_s_* Trapezoid	[60]	(29)	*T_a_,VPD, u, T_s_,VI, R_n_, G*	1) Dry and wet edges are linear lines and vary linearly with *VI* 2) *EF* varies linearly with *T_s_* for a given *VI*.	Whole range of *VI* and soil moisture in the scene of interest is not required;	1) Uncertainty in the determination of dry and wet edges; 2) A lot of ground - based measurements are needed.
SEBI	[81]	(17)	*T_pbl_, h_pbl_, u, T_s_, R_n_, G*	1) Dry limit has a zero surface *ET*; 2) Wet limit evaporates potentially.	Directly relating the effects of *T_s_* and *r_a_* on *LE*.	Ground-based measurements are needed.
SEBAL	[33]	(26)	*u, z_a_,T_s_, VI, R_n_, G*	1) Linear relationship between *T_s_* and *dT*; 2) ET of the driest pixel is 0; 3) *ET_wet_* is set to the surface available energy.	1) Minimum ground measurements 2) Automatic internal calibration; 3) Accurate atmospheric corrections are not needed	1) Applied over flat surfaces; 2) Uncertainty in the determination of anchor pixels.
S-SEBI	[34]	(25)	*T_s_, α_s_, R_n_, G*	1) *EF* varies linearly with *T_s_* for a given surface albedo. 2) *T_s,max_* corresponds to the minimum *LE*. 3) *T_s,min_* corresponds to the maximum *LE*.	No ground-based measurements are needed	Extreme temperatures have to be location specific.
SEBS	[2]	(20)	*T_a_, z_a_, u,T_s_, R_n_, G*	1) At the dry limit, ET is set to 0; 2) At the wet limit, ET takes place at potential rate.	1) Uncertainty in SEBS from *T_s_* and meteorological variables can be limited and reduced; 2) Computing explicitly the roughness height for heat transfer instead of using fixed values.	1) Too many parameters are required 2) Solution of the turbulent heat fluxes is relatively complex.
METRIC	[36] [105]	(26)	*u, z_a_,T_s_, VI, R_n_, G*	1) For the hot pixel, ET is equal to zero 2) For the wet pixel, *LE* is set to 1.05*ET_r_*.	Same as SEBAL but surface slope and aspect can be considered.	Uncertainty in the determination of anchor pixels.
TSM	[37]	Soil and canopy energy budgets	*u, z_a_ T_a_,T_s_, T_c_, F_r_* or *LAI, R_n_, G*	1) Fluxes of soil surfaces are in parallel or in series with fluxes of canopy leaves; 2) Priestly-Taylor Equation is employed to give the first-guess of canopy transpiration	1) Effects of view geometry are taken into account; 2) Empirical corrections for the ‘excess resistance’ are not needed;	1) Many ground measurements are needed. 2) Component temperatures of soil and vegetation are required.
TSTIM/ALEXI	[137]	Soil and canopy energy budgets	*u, z_a_ dTs, F_r_* or *LAI, R_n_, G*.	Surface temperature changes linearly with the time during the morning hours of the sensible heating	Errors due to atmospheric corrections and surface emissivity specification are significantly reduced;	Determination of an optimal pair of thermal observation times for the linear rise in sensible heating is needed.

## Appendix

Nomenclature used in this paper:

**Table:**

*C_p_*	Specific heat of air at constant pressure	J/(m·K)
*C_rad_*	Correction coefficient used in sloping terrain	-
*d*	Zero plane displacement height	m
*dT*	Surface-air temperature difference	K
*dT_dry_*	Surface-air temperature difference at dry pixel	K
*dT_wet_*	Surface-air temperature difference at wet pixel	K
*dTs*	Surface temperature difference of two times in the morning	K
*D*	Day of year	day
*EF*	Evaporative fraction	-
*EF_r_*	Relative evaporative fraction	-
*ET*	Evapotranspiration	mm/h
*ET_i_*	Instantaneous ET	mm/h
*ET_r_*	Reference ET (over the standardized 0.5 m tall alfalfa)	mm/h
*ET_d_*	Cumulative daily ET	mm/d
*ET_r_d_*	Cumulative daily reference ET	mm/d
*ET_r_F*	Reference ET fraction	-
*F_r_*	Fractional vegetation cover	-
*f (θ)*	Fraction of canopy in the field of view of the radiometer	-
*g*	Acceleration due to gravity of the earth	m/s^2^
*G*	Soil heat flux density	W/m^2^
*h_pbl_*	Height of the PBL	m
*H*	Sensible heat flux	W/m^2^
*H_dry_*	Sensible heat flux at dry limit	W/m^2^
*H_wet_*	Sensible heat flux at wet limit	W/m^2^
*k*	Von Karman's constant	-
*L*	Latent heat of vaporizaiton	J/kg
*LE*	Latent heat flux density	W/m^2^
*LE_d_*	Daily ET	mm/d
*LE_p_*	Potential ET	W/m^2^
*LE_wet_*	Latent heat flux at wet limit	W/m^2^
*N*	Daylight period between sunrise and sunset	h
*r_a_*	Stability-corrected aerodynamic resistance to heat transfer between surface and reference height	s/m
*r_a,max_*	Maximum aerodynamic resistance to sensible heat transfer	s/m
*r_a,min_*	Minimum aerodynamic resistance	s/m
*r_e_*	Excess resistance	s/m
*r_rc_*	Effective radiometric-convective resistance	s/m
*r_s_*	Soil-surface resistance	s/m
*R_s_*	Incoming shortwave solar radiation	W/m^2^
*t*	Duration time starting at sunrise	h
*T_a_*	Air temperature measured at a reference height	K
*T_c_*	Vegetation canopy temperature	K
*T_0_*	Soil surface temperature	K
*T_pbl_*	Average planetary boundary layer temperature	K
*T_RAD_(θ)*	Directional radiometric surface temperature	K
*T_B_(θ)*	brightness temperature	K
*T_s,max_*	Maximum surface temperature	K
*T_s,min_*	Minimum surface temperature	K
*u*	Wind speed	m/s
*u**	Friction velocity	m/s
*z_a_*	Measurement height of wind speed and air temperature	m
*z_oh_*	Surface roughness length for heat transfer	m
*z_om_*	Surface roughness length for momentum transfer	m
*α_s_*	Surface shortwave albedo	-
*γ*	Psychrometric constant	kPa/°C
*Δ*	Slope of saturated vapor pressure as a function of Ta	kPa/°C
*ε_a_*	Atmospheric emissivity	-
*ε_s_*	Surface emissivity	-
*λ*	Geographical latitude (expressed in decimal degrees)	degrees
*Λ*	Monin-Obukhov length	m
*ρ*	Density of a certain entity	kg/m^3^
*ρ_w_*	Density of water	kg/m^3^
*σ*	Stefan-Boltzman constant (5.67×10^-8^)	W/(m^2^K^4^)
*ϕ*	Combined-effects parameter in equations (28) and (29)	-
*θ*	View zenith angle	degrees
*Ψ_1_*	Stability correction function for momentum transfer	-
*Ψ_2_*	Stability correction function for heat transfer	-
